# Apolipoprotein E4 Causes Age- and Sex-Dependent Impairments of Hilar GABAergic Interneurons and Learning and Memory Deficits in Mice

**DOI:** 10.1371/journal.pone.0053569

**Published:** 2012-12-31

**Authors:** Laura Leung, Yaisa Andrews-Zwilling, Seo Yeon Yoon, Sachi Jain, Karen Ring, Jessica Dai, Max Mu Wang, Leslie Tong, David Walker, Yadong Huang

**Affiliations:** 1 Gladstone Institute of Neurological Disease, San Francisco, California, United States of America; 2 Gladstone Institute of Cardiovascular Disease, San Francisco, California, United States of America; 3 Department of Neurology, University of California San Francisco, San Francisco, California, United States of America; 4 Department of Pathology, University of California San Francisco, San Francisco, California, United States of America; 5 Biomedical Sciences Graduate Program, University of California San Francisco, San Francisco, California, United States of America; University of Kentucky, United States of America

## Abstract

Apolipoprotein (apo) E4 is the major genetic risk factor for Alzheimer's disease (AD). ApoE4 has sex-dependent effects, whereby the risk of developing AD is higher in apoE4-expressing females than males. However, the mechanism underlying the sex difference, in relation to apoE4, is unknown. Previous findings indicate that apoE4 causes age-dependent impairments of hilar GABAergic interneurons in female mice, leading to learning and memory deficits. Here, we investigate whether the detrimental effects of apoE4 on hilar GABAergic interneurons are sex-dependent using apoE knock-in (KI) mice across different ages. We found that in female apoE-KI mice, there was an age-dependent depletion of hilar GABAergic interneurons, whereby GAD67- or somatostatin-positive–but not NPY- or parvalbumin-positive–interneuron loss was exacerbated by apoE4. Loss of these neuronal populations was correlated with the severity of spatial learning deficits at 16 months of age in female apoE4-KI mice; however, this effect was not observed in female apoE3-KI mice. In contrast, we found an increase in the numbers of hilar GABAergic interneurons with advancing age in male apoE-KI mice, regardless of apoE genotype. Moreover, male apoE-KI mice showed a consistent ratio of hilar inhibitory GABAergic interneurons to excitatory mossy cells approximating 1.5 that is independent of apoE genotype and age, whereas female apoE-KI mice exhibited an age-dependent decrease in this ratio, which was exacerbated by apoE4. Interestingly, there are no apoE genotype effects on GABAergic interneurons in the CA1 and CA3 subregions of the hippocampus as well as the entorhinal and auditory cortexes. These findings suggest that the sex-dependent effects of apoE4 on developing AD is in part attributable to inherent sex-based differences in the numbers of hilar GABAergic interneurons, which is further modulated by apoE genotype.

## Introduction

Alzheimer's disease (AD) is a common age-dependent neurodegenerative disease characterized by progressive and irreversible cognitive decline [1], [2]. Epidemiological studies that focus on disease prevalence as a measure of risk have presented mixed results in regards to sex susceptibility to AD [3], [4], [5], which may be confounded by the higher longevity of woman compared to men. However, reports looking at age-specific incidences of AD show a clear sex-specific difference [6], [7], [8], [9], with women possessing a higher overall risk for developing AD. Moreover, sex discrepancies in the pathological features of AD and its relationship to behavioral disturbances indicate a pathophysiological basis for the differences [10]. Each unit increase in AD pathology was associated with a 3-fold increase in the clinical manifestation of AD among men, compared to more than 20-fold increase in women [11].

Apolipoprotein (apo) E4, found in 65–80% of late onset sporadic and familial AD cases, is the predominant genetic risk factor, and its expression increases the occurrence and lowers the age of onset of AD in a gene dose-dependent manner [12], [13], [14]. Epidemiological studies have further indicated a sex-specific association with respect to the apoE4 allele, whereby women expressing this allele have a higher risk of being affected by AD than men, regardless of longevity and disease mortality factors [14], [15], [16], [17], [18], [19]. Rodent studies have recapitulated this finding, demonstrating that female mice expressing neuron-specific enolase (NSE) apoE are more susceptible to apoE4-induced impairments of spatial learning and memory than their male counterparts [20], [21], [22]. Sex differences in cognitive function have been attributed to hormone-induced differences in hippocampal morphology and function [23], [24], [25], and accordingly, manipulations of hormone levels or its associated receptors modify cognitive ability. Blockage of androgen receptors in male NSE-apoE4 mice impairs spatial learning and memory [26]. Conversely, testosterone, DHT, and selective androgen receptor modulators ameliorate cognitive deficits in female NSE-apoE4 mice [26], [27]. However, the underlying pathogenic mechanism contributing to sex discrepancy in AD risk in relation to apoE4 is unknown.

We previously demonstrated that apoE4 impairs hilar GABAergic interneurons in an age-dependent manner in female human apoE knock-in (KI) mice, and that this impairment precedes learning and memory deficits [28]. Moreover, treating female apoE4-KI mice with pentobarbital, a GABA_A_ receptor potentiator, rescued the apoE4-induced cognitive deficits [28], suggesting a role for reduced GABA signaling in this process. Optogenetic manipulations of hilar GABAergic interneurons confirmed that functional inhibition of this specific neuronal population results in learning and memory deficits [29]. Based on these findings, we hypothesize that the apoE4-associated sex discrepancies in AD risk are a reflection of differences in the impairment of hippocampal GABAergic interneurons. In the present study, we investigated whether changes in hilar GABAergic interneurons contribute to cognitive decline in relation to age, sex, and apoE isoforms in mice.

## Materials and Methods

### Animals

Male and female human apoE3-KI and apoE4-KI mice on a C57BL/6 genetic background [30], [31] were from Taconic (Hudson, NY). Wild-type mice on a C57BL/6 genetic background were from Jackson Laboratory (Bar Harbor, ME). Studies were conducted on male and female mice at 1, 3, 6, 12, and 16 months of age. Female mice were housed together in the absence of exposure to male pheromones to synchronize estrous cycles [32]. Equal numbers of mice for each apoE genotype at each age were used. All animal experiments were performed in accordance with the National Institutes of Health, University of California San Francisco, and Gladstone institutional guidelines.

### BrdU Injections

To label newly generated GABAergic interneurons, male and female apoE3-KI and apoE4-KI mice (14.5 months of age) received twice daily intraperitonal injections of BrdU (100 mg/kg body weight, 6 hours apart; Sigma) in their home cages for 14 days. At 4 weeks after the last BrdU injection (at 16 months of age), the brains were perfused with 0.9% NaCl, collected, and immunostained as described below.

### Immunostaining and image collection

Brains were collected after a 1-min transcardial perfusion with 0.9% NaCl. One hemibrain from each mouse was fixed in 4% paraformaldehyde, followed by cryoprotection in 30% sucrose. Coronal sections (30 µm-thick) were cut continuously throughout the entire hippocampus with a microtome and divided into subseries of every tenth section, yielding 8–9 sections containing the hippocampus in each animal [33], [34]. One subseries of every tenth section was immunostained with the following primary antibodies: monoclonal mouse anti-glutamic acid decarboxylase-67 (GAD67, 1∶1000 for DAB; Millipore), rat anti-somatostatin (1∶100 for DAB; Millipore), rabbit anti-neuropeptide Y (NPY, 1∶8000 for DAB; Sigma), rabbit anti-parvalbumin (1∶5000 for DAB; Swant), mouse anti-NeuN (1∶1000 for DAB; Millipore), rabbit anti-calretinin (1∶750 for DAB; Millipore), rabbit anti-GABA (1∶2000 for immunofluorescence; Sigma), or mouse anti-BrdU (1∶200 for immunofluorescence; Chemicon). Primary antibodies were detected with the following secondary antibodies: biotinylated donkey anti-mouse IgG, biotinylated rabbit anti-rat IgG, or biotinylated goat anti-rabbit IgG (all 1∶250; Vector Laboratories) for DAB staining, or Alexa Fluor 488-labeled donkey anti-rabbit IgG (1∶500; Invitrogen), and Alexa Fluor 594-labeled donkey anti-mouse IgG (1∶500; Invitrogen) for immunofluorescence. GAD67 immunohistochemistry was elected over GAD65 labeling because of its relative specificity to the soma over axonal boutons [35], [36], [37]. All brain sections for each GABAergic marker were processed in parallel using the same batches of solutions to minimize variability in immunohistochemical labeling conditions. Specificity of the immune reaction for each antibody was controlled by omitting the primary antiserum. Stained sections were examined with a Leica epifluorescence microscope (Germany) or a Radiance 2000 laser-scanning confocal system (Bio-Rad) mounted on a Nikon Optiphot-2 microscope (Japan).

### Quantitative analyses of immunostained brain sections

GABAergic interneuron numbers were quantified by design-based stereology, which allows for estimates of cell number by assuming that a few plane sections are representative of the entire brain [38], [39], [40]. For male apoE-KI mice, all GAD67-, somatostatin-, neuropeptide Y-, and parvalbumin-positive interneuron data are new. For female apoE-KI mice, all neuropeptide Y- and parvalbumin-positive interneuron data are also new. For female GAD67- and somatostatin-positive interneuron data, we combined a few newly collected mouse brains with previously collected and published mouse brains [28], with permission from the *Journal of Neuroscience*, and re-stained brain sections for quantification and analysis. GAD67-, somatostatin-, NPY-, and parvalbumin-positive cells in the hilus of the dentate gyrus were counted in every 10^th^ serial coronal section throughout the rostrocaudal extent of the hippocampus by an investigator blinded to the age, sex, and genotype. The hilus is defined as the polymorphic nuclear region between the inner border of the granule cell layer and an imaginary connection between the ends of both granule cell blades except for the interposed layer of CA3 pyramidal neurons [41]. Stained cells with clear cellular boundaries were considered positive, although positive cells touching the granular layer were rejected. “Cells” that were stained very lightly and/or had irregular shapes were excluded from quantitation. Results are presented as the total number of positive cells counted per hemibrain, multiplied by 2 (for both hemibrains), and then by 10 (for every 10^th^ serial section) [28]. To determine hilar specificity in changes of GABAergic interneuron numbers, GAD67-positive cells were also quantified in the auditory cortex, entorhinal cortex, and the strata oriens, pyramidale, and radiatum of CA3 and CA1. The densities of GAD67 immunoreactivity in the hilus and of parvalbumin-positive processes in the molecular layer were analyzed with ImageJ software. For measurement of GAD67 and parvalbumin density, the total region of interest was manually outlined and averaged densities normalized to area were acquired. The mean pixel density of at least seven pictures per brain was averaged per mouse.

### Morris water maze test

The water maze pool (diameter 122 cm) contained opaque water (22–23°C) with a platform 10 cm in diameter. The platform was submerged 1.5 cm from the surface during the hidden platform sessions [20], [42] and marked with a black-and-white-striped mast (15 cm high) during the cued training sessions. Mice at 12 or 16 months of age were trained to locate the hidden platform (hidden days 1–5) and the cued platform (visible days 1–3) in two daily sessions spaced by 3.5 hours, each consisting of two 60-second trials (hidden and cued training) with a 15 min interval [28]. The platform location remained constant throughout the hidden platform sessions but was changed for each cued platform session. Entry points were changed semi-randomly between trials. Escape latency is noted as the time taken to locate the hidden platform. Swim speed is assessed as the path length to the platform divided by latency. 24 and 72 hours after the last hidden platform training, we performed a 60-second probe trial with the platform removed. Entry points for the probe trial were in the northwest quadrant, and the target quadrant was the southeast quadrant. Performance was monitored with an EthoVision video-tracking system (Noldus Information Technology). The data are presented as the percentage of time spent in a given location, which compares the average time spent in the target quadrant to the average time spent in each of the other three quadrants.

### Correlation of hilar GABAergic interneuron numbers with spatial learning abilities

Average escape latency in hidden platform days 1−5 of the Morris water maze in 16-month-old male and female apoE-KI mice was compared to the number of immunopositive hilar interneurons in the corresponding mouse (n = 6−12). The coefficient of determination R^2^ was used to determine the extent of correlation between the two variables.

### Statistical Analysis

All values are expressed as mean ± SEM. Statistical analyses were performed with GraphPad Prism version 4.0 software. Latencies of all groups of mice in the Morris water maze were analyzed and compared by repeated measures ANOVA and Bonferroni *post-hoc* test. Differences between the means for histochemical quantifications were assessed by unpaired *t*-test or two-factor ANOVA followed by a Bonferroni *post-hoc* test. A *p*-value of <0.05 was considered to be statistically significant. Statistical values are denoted as follows: * *p*<0.05, ** *p*<0.01, *** *p*<0.001.

## Results

### Spatial learning and memory deficits in apoE4-KI mice are sex dependent

**Figure 1:**
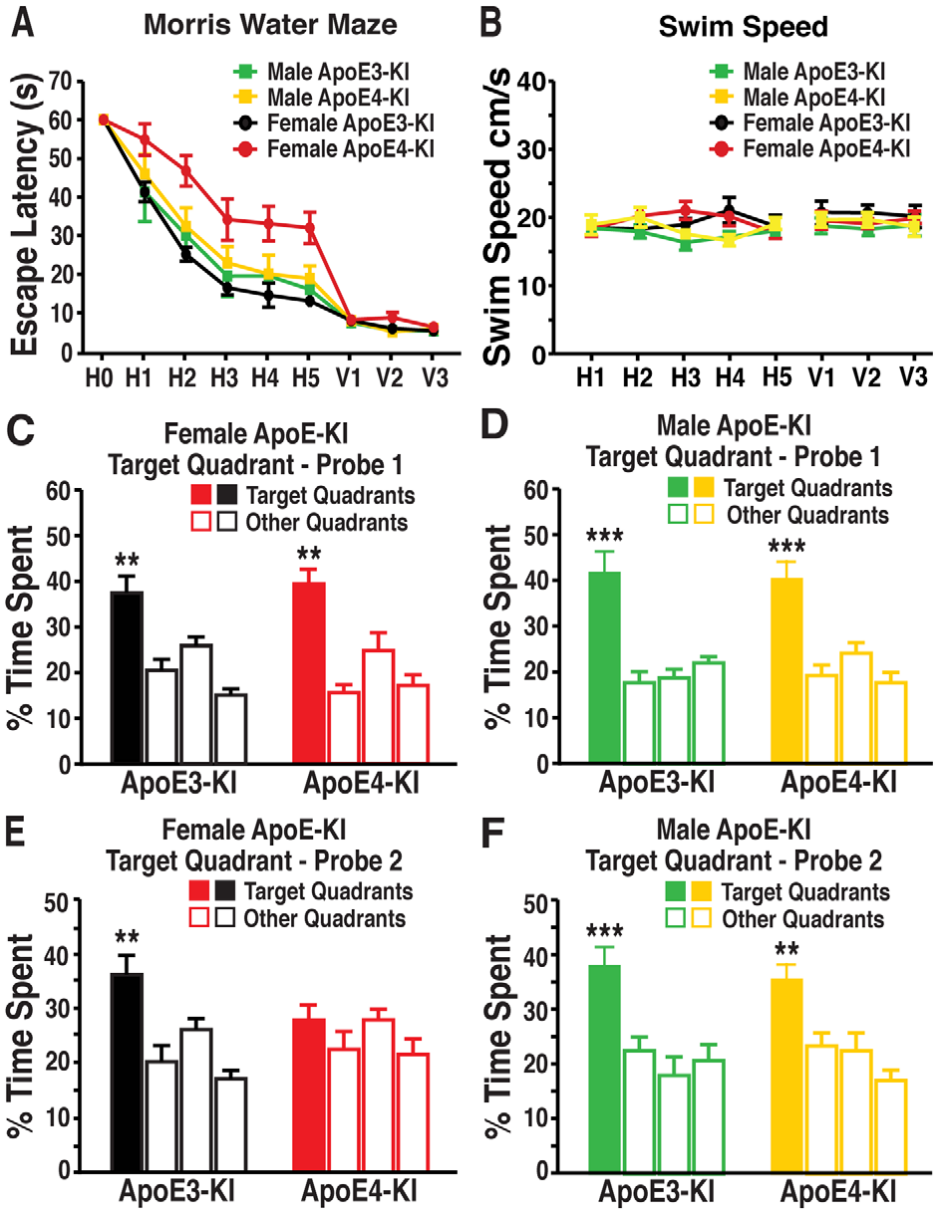
Aged female mice show apoE4-induced spatial learning and memory deficits, but male mice do not. ***A***, 16-month old male and female apoE3-KI or apoE4-KI mice (n = 10−12 mice per group) were tested in the Morris water maze. Points represent averages of daily trials. H, hidden platform day (2 trials/session, 2 sessions/day); H0, first trial on H1; V, visible platform day (2 trials/session, 2 sessions/day). Escape latency (*y*-axis) indicates time to reach the target. In the hidden platform days, latencies of all groups of mice were analyzed and compared by repeated measures ANOVA and Bonferroni *post-hoc* test. Female apoE4-KI mice learned significantly slower than female apoE3-KI mice (repeated-measures ANOVA and Bonferroni *post-hoc* test, *p*<0.01) [28]. Male apoE3-KI and apoE4-KI mice performed at a similar level to female apoE3-KI mice. ***B***, Swim speed was not different among the various groups of mice. ***C, D***, Probe 1 trials of female (***C***, n = 10−12) and male (***D***, n = 7−9) apoE3-KI or apoE4-KI mice were performed 24 h after the last hidden day platform training. ***E, F***, Probe 2 trials of female (***E***, n = 10−12) and male (***F***, n = 7−9) apoE3-KI or apoE4-KI mice were performed 72 h after the last hidden day platform training. Percentage time spent in the target quadrant versus the time spent in any of the three non-target quadrants differed in all groups except for female apoE4-KI mice. **p<0.01, ***p<0.001 (*t*-test).

To assess the effects of sex and apoE4 on spatial learning and memory, male and female apoE-KI mice were first tested at 12 months of age in the Morris water maze assay. All apoE-KI mice, independent of sex or apoE isoform, exhibited normal cognitive function at this age, as demonstrated by an equal ability to learn and remember the location of the hidden platform (Fig. S1 *A*−*F*). We subsequently examined apoE-KI mice at 16 months of age in the Morris water maze. As reported in our previous publication [28], female apoE3-KI mice quickly learned to locate the hidden platform, whereas female apoE4-KI mice exhibited greater response latencies, suggesting deficits in learning the task (repeated-measures ANOVA; *F*_(4,67)_ = 9.217, *p*<0.01; post-hoc comparison; apoE4-KI vs apoE3-KI, *t*_(20)_ = 3.521, *p*<0.01) (Fig. 1*A*). Memory was also compromised in the female apoE4-KI mice, as they were unable to remember the location of the hidden platform in the second probe trial (Fig. 1*E*), although they remembered as well as apoE3-KI mice in the first probe trial (Fig. 1*C*). In contrast, both male apoE3-KI and apoE4-KI mice performed equally well in the hidden platform (repeated-measures ANOVA, *p*>0.05; *post-hoc* comparisons: apoE3-KI vs apoE4-KI, *p*>0.05) and probe trials (Figs. 1*A, D, F*), suggesting normal learning and memory function. Swim speeds did not differ among the four groups of mice (Fig. 1*B*). Thus, learning and memory deficits associated with apoE4 are sex dependent, with females being more susceptible to apoE4′s detrimental effects.

### Female but not male apoE4-KI mice demonstrate age-dependent impairments in the expression of specific subsets of hilar GABAergic interneurons

**Figure 2:**
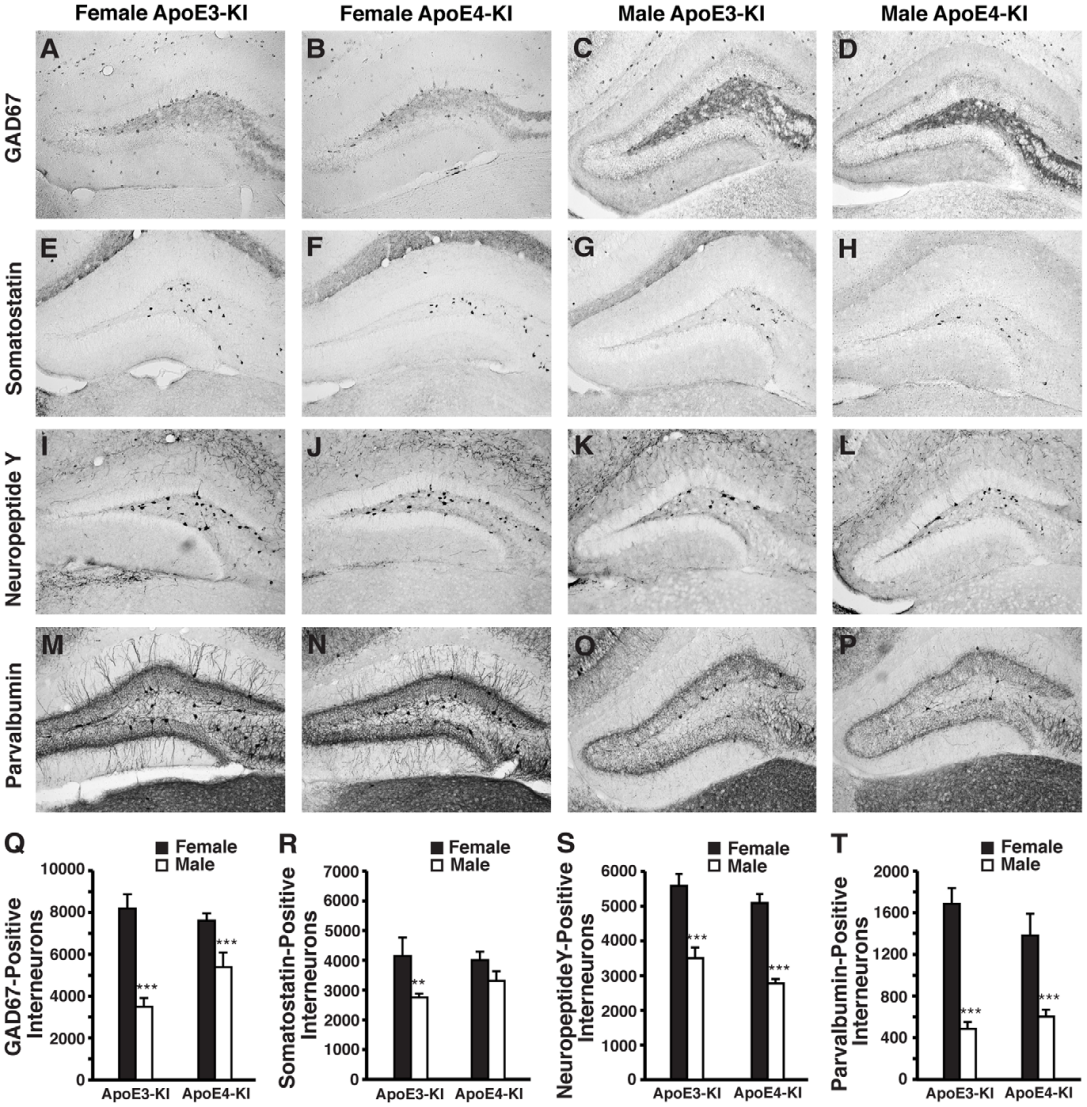
ApoE-KI mice exhibit basal sex differences in hilar GABAergic interneurons at 1-month of age. ***A–D***, Representative images (200x) of anti-GAD67-immunostained sections of the hilus of female apoE3-KI (***A***), female apoE4-KI (***B***), male apoE3-KI (***C***), and male apoE4-KI (***D***) mice at 1 month of age. ***E–H***, Representative images (200x) of anti-somatostatin-immunostained sections of the hilus of female apoE3-KI (***E***), female apoE4-KI (***F***), male apoE3-KI (***G***), and male apoE4-KI (***H***) mice at 1 month of age. ***I–L***, Representative images (200x) of anti-NPY-immunostained sections of the hilus of female apoE3-KI (***I***), female apoE4-KI (***J***), male apoE3-KI (***K***), and male apoE4-KI (***L***) mice at 1 month of age. **M–P**, Representative images (200x) of anti-parvalbumin-immunostained sections of the hilus of female apoE3-KI (***M***), female apoE4-KI (***N***), male apoE3-KI (***O***), and male apoE4-KI (***P***) mice at 1 month of age. ***Q–T***, Quantification of hilar GABAergic interneurons positive for GAD67 (***Q***), somatostatin (***R***), neuropeptide Y (***S***), or parvalbumin (***T***) in female and male apoE-KI mice at 1 month of age (n = 6–12 per group). Results in histograms are presented as the total number of positive cells counted per brain. Male apoE-KI mice have significantly fewer hilar GABAergic interneurons than female apoE-KI mice at 1 month of age by *t-*test, **p<0.01; ***p<0.001.

**Figure 3:**
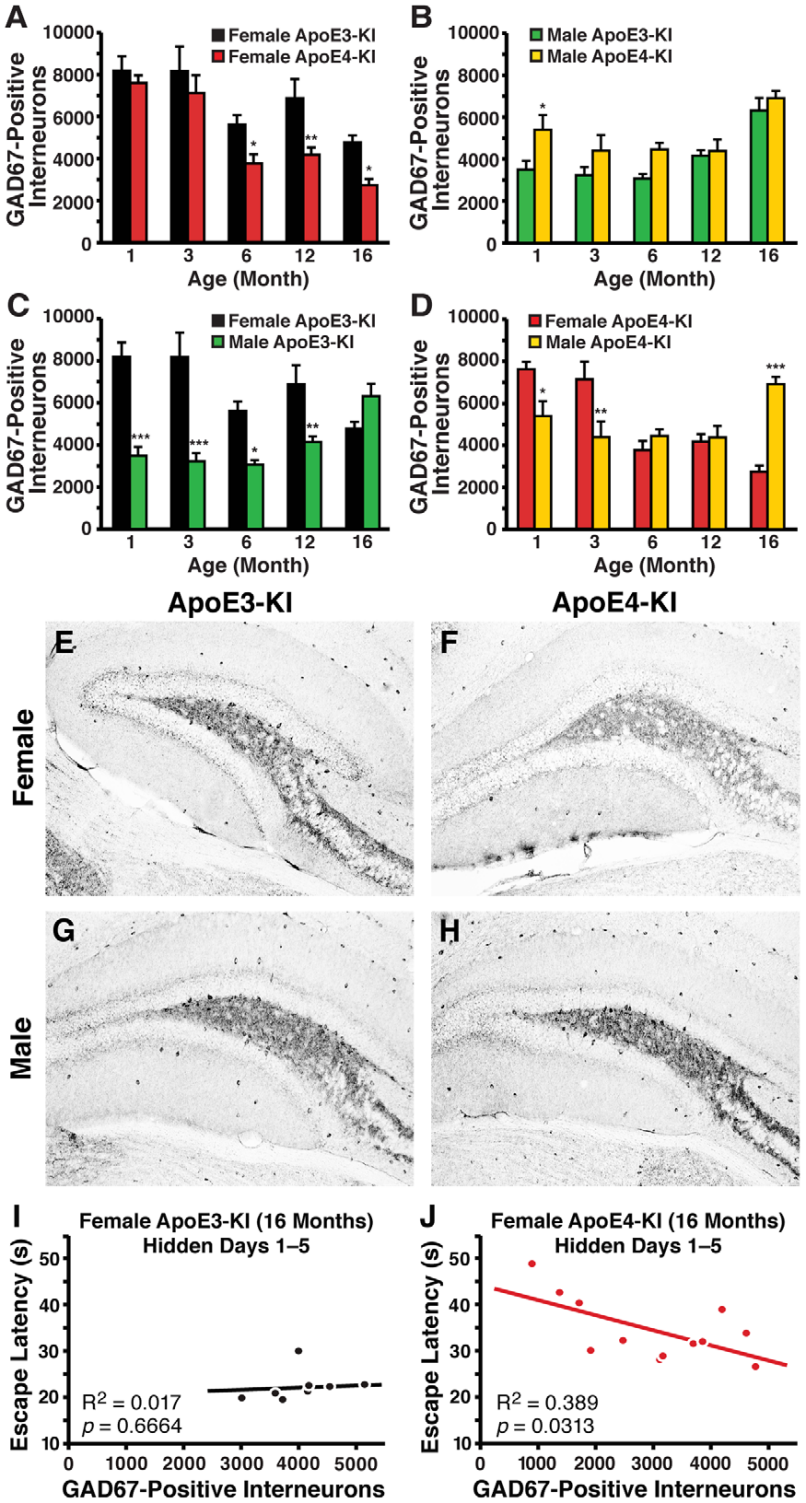
GAD67-immunopositive hilar GABAergic interneuron numbers change as a function of age, sex, and apoE isoforms. ***A, B***, Quantification of hilar GABAergic interneurons positive for GAD67 in female (***A***) or male (***B***) apoE-KI mice at 1, 3, 6, 12, and 16 months of age (n = 6−12 mice per group). Results in histograms are presented as the total number of positive cells counted per brain. Female apoE-KI mice show an apoE isoform-dependent and age-dependent effect, but no interaction between the two variables was observed by 2-way ANOVA [28]. *p<0.05; **p<0.01 in female apoE4-KI mice compared to female apoE3-KI mice at the same age (*post-hoc* Bonferroni test). Male apoE-KI mice also show an apoE isoform–dependent effect, and further exhibit an interaction between the two variables by 2-way ANOVA, *p<0.05 (*post-hoc* Bonferroni test). ***C, D***, Quantification of GAD67-positive hilar interneurons in female and male apoE3-KI (***C***) or apoE4-KI (***D***) mice at 1, 3, 6, 12, and 16 months of age (n = 6−12 mice per group). ApoE3-KI mice show a sex-dependent effect, with interaction between sex and age by 2-way ANOVA. ApoE4-KI mice show an age-dependent, but sex-independent, effect with interaction between the two variables by 2-way ANOVA. *p<0.05; **p<0.01; ***p<0.001 in male apoE-KI mice compared to their female counterparts (*post-hoc* Bonferroni test). ***E–H***, Representative images (200x) of anti-GAD67-immunostained sections of the hilus of female apoE3-KI (***E***), female apoE4-KI (***F***), male apoE3-KI (***G***), and male apoE4-KI (***H***) mice at 16 months of age. ***I, J***, Escape latency in hidden platform days 1−5 correlated inversely with the number of GAD67-positive hilar interneurons in female apoE4-KI mice (***J***, n = 12) but not female apoE3-KI mice (***I***, n = 10) at 16 months of age [28].

Because learning and memory impairments in female apoE4-KI mice did not appear until 16 months of age, a temporal analysis on the effects of sex and apoE genotype on GABAergic interneurons was performed. Hilar GABAergic interneuron numbers were quantified in male and female apoE3-KI and apoE4-KI mice at 1, 3, 6, 12, and 16 months of age. Our initial study involved the analysis of GAD67-positive and somatostatin-positive interneurons in the hilus of the dentate gyrus, as they exhibited significant age-dependent impairments in female apoE4-KI mice compared to female apoE3-KI mice [28]. Interestingly, we observed basal sex-specific differences that were independent of apoE isoform. At 1 month of age, female apoE-KI mice exhibited 50–100% more GAD67-positive interneurons than male apoE-KI mice (Fig. 2*A–D, Q*). However, GAD67 immunoreactivity, including that in neuronal processes, in the hilus was higher in male apoE-KI mice compared to females (*t*-test; apoE3-KI: *p*<0.001 males versus females; apoE4-KI: *p*<0.01 males versus females) (Figs. 2*A–D and* S2*A*), which might be attributed to compensatory increases in GAD67 expression in each hilar GABAergic interneuron in response to lower numbers of total inhibitory neurons in males. Analysis of aged mice revealed that female apoE4-KI mice had significantly reduced numbers of hilar GAD67-positive interneurons compared to female age-matched apoE3-KI mice starting at 6 months of age (*post-hoc* Bonferroni; *p*<0.05 at 6 months, *p*<0.01 at 12 months, *p*<0.05 at 16 months) (Fig. 3*A, E, F*). These effects were age- and apoE genotype-dependent (ANOVA: age, *F*_(4,67)_ = 18.36, *p*<0.005; apoE genotype, *F*_(1,67)_ = 18.71, *p*<0.005; interaction, *F*_(4,67)_ = 0.9817, *p*>0.05) and remained significant even during the age-related neuronal loss present in both genotypes. Moreover, the loss of GAD67-positive interneurons correlated with spatial learning deficits in female apoE4-KI mice at 16 months of age (R^2^ = 0.389, *p* = 0.0313) (Fig. 3*J*), but not in female apoE3-KI mice (R^2^ = 0.017, *p* = 0.6664) (Fig. 3*I*). At this age, all female apoE3-KI mice had >2500 hilar GABAergic interneurons (Fig. 3*I*), whereas some of the female apoE4-KI mice had <2500 and had greater learning deficits (Fig. 3*J*). Interestingly, none of the female apoE4-KI mice at 12 months of age had <2500 hilar GABAergic interneurons, and none of them had learning deficits at 12 months. Accordingly, there was no correlation between hilar GAD67-positive interneuron numbers and spatial learning in female apoE3-KI or apoE4-KI mice at 12 months of age (data not shown). Thus, 2500 may be the threshold number of hilar GABAergic interneurons that determines normal versus impaired learning performance of female apoE-KI mice in the Morris water maze, as suggested previously [*28*].

**Figure 4:**
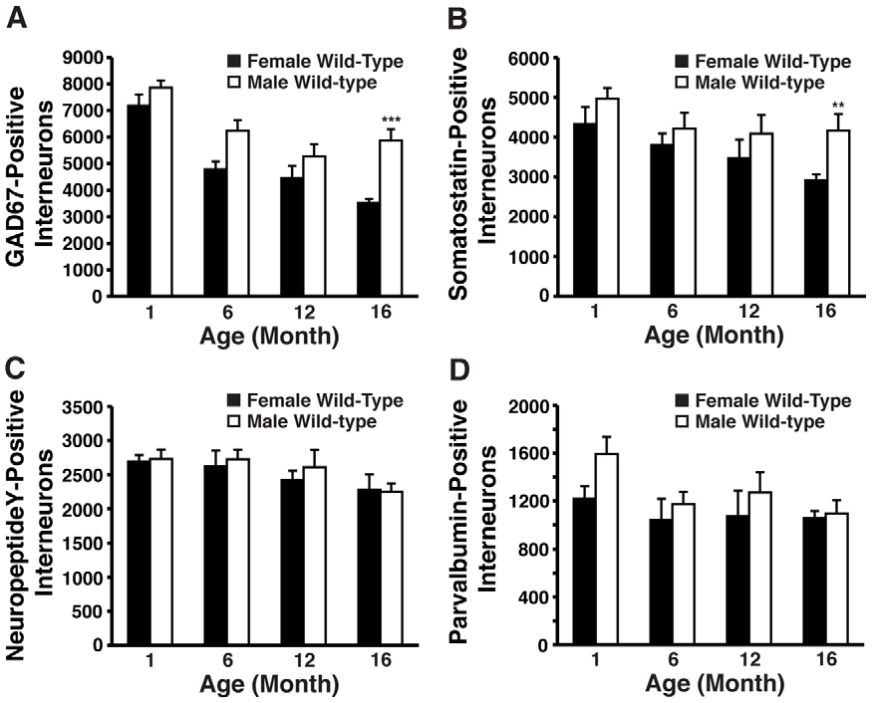
Age- and sex-dependent effects on numbers of hilar GABAergic interneurons in wild-type mice. ***A–D***, Hilar GABAergic interneurons positive for GAD67 (***A***), somatostatin (***B***), neuropeptide Y (***C***), and parvalbumin (***D***) in female and male wild-type mice at 1, 6, 12, and 16 months of age (n = 6−8 mice per group). Results in histograms are presented as the total number of positive cells counted per brain. **p<0.01; ***p<0.005 (*post-hoc* Bonferroni test).

Male apoE4-KI mice had significantly greater numbers of GAD67-positive hilar interneurons than male apoE3-KI mice at 1 month of age (*t-*test, *p*<0.01) (Fig. 3*B*). However, this difference in the numbers of GAD67-positive interneurons between male apoE3-KI and apoE4-KI mice became negligible beginning at 3 months and persisted through 16 months of age (*post-hoc* Bonferroni, *p*>0.05) (Fig. 3*B, G, H*). Interestingly, male apoE-KI mice exhibited an age- and apoE genotype–dependent increase in hilar GAD67-positive interneurons (ANOVA: age, *F*_(4,51)_ = 8.438, *p*<0.005; apoE genotype, *F*_(1,51)_ = 12.59, *p*<0.005; interaction, *F*_(4,51)_ = 3.534, *p*<0.05) (Fig. 3*B, G, H* *)*. We found no correlation between spatial learning properties and the numbers of GAD67-positive hilar interneurons in male apoE-KI mice at 16 months of age (R^2^ = 0.0008, *p*>0.05 for male apoE3-KI mice; R^2^ = 0.0014, *p*>0.05 for male apoE4-KI mice) (data not shown). Among apoE3-KI mice, significant sex-dependent effects on GAD67-positive hilar interneurons were noted (ANOVA: age, *F*_(4,50)_ = 1.844, *p*>0.05; sex, *F*_(1,50)_ = 46.59, *p*<0.001; interaction, *F*_(4,50)_ = 8.725, *p*<0.001), with male apoE3-KI mice exhibiting significantly less GAD67-positive hilar interneurons than age-matched female apoE3-KI mice from 1 month of age through 12 months of age (*post-hoc* Bonferroni; *p*<0.001 at 1 month, *p*<0.001 at 3 months, *p*<0.05 at 6 months, *p*<0.01 at 12 months) (Fig 3*C*). Although a sex-dependent effect was not evident among apoE4-KI mice with aging (ANOVA: age, *F*_(4,55)_ = 8.185, *p*<0.001; sex, *F*_(1,55)_ = 0.001, *p*>0.05; interaction, *F*_(4,55)_ = 14.96, *p*<0.001), sex differences in numbers of GAD67-positive interneurons were detected at various ages (*post-hoc* Bonferroni; *p*<0.05 at 1 month, *p*<0.0 at 3 months, *p*<0.001 at 16 months) (Fig. 3*D*). An age-dependent decrease in hilar GAD67-positive interneurons was also apparent in female wild-type mice (ANOVA: age, *F*_(3,47)_ = 22.64, *p*<0.001), with total numbers at 16 months of age being significantly reduced compared to male wild-type mice (*post-hoc* Bonferroni; *p*<0.001) (Fig. 4*A*).

**Figure 5:**
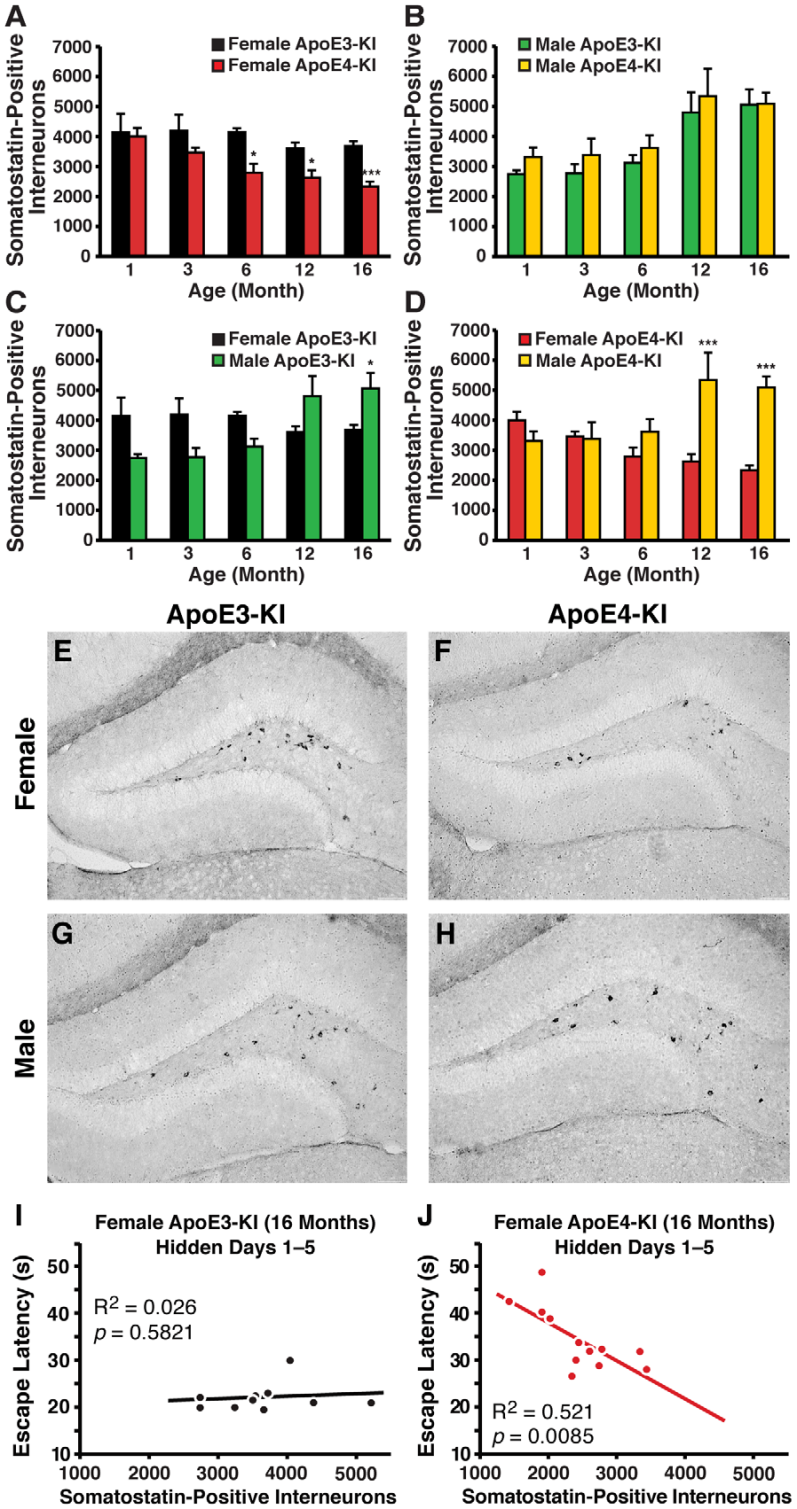
Age- and sex-dependent effects on numbers of somatostatin-immunopositive hilar GABAergic interneurons in apoE-KI mice. ***A, B***, Histograms showing the total number of somatostatin-positive hilar GABAergic interneurons in female (***A***) or male (***B***) apoE3-KI or apoE4-KI mice at 1, 3, 6, 12, and 16 months of age (n = 6−12 mice per group). Female apoE-KI mice show an apoE isoform-dependent and age-dependent effect, but no interaction between the two variables by 2-way ANOVA [28]. Male apoE-KI mice demonstrate an age-dependent effect, but no apoE isoform–dependent effects. No interaction was detected between the two variables. *p<0.05; ***p<0.005 in female apoE4-KI mice compared to female apoE3-KI mice at the same age (*post-hoc* Bonferroni test). ***C, D***, Quantification of somatostatin-positive hilar GABAergic interneurons in female and male apoE3-KI (***C***) or apoE4-KI (***D***) mice at 1, 3, 6, 12, and 16 months of age (n = 6−12 mice per group). ApoE3-KI mice have age-dependent and sex-independent effects, whereas apoE4-KI mice show an age-independent and sex-dependent effect. Both apoE genotypes show an interaction between age and sex by 2-way ANOVA. *p<0.05; ***p<0.001 in male apoE-KI mice compared to female apoE-KI mice at the same age (*post-hoc* Bonferroni test). ***E–H***, Representative images (200x) of somatostatin-immunostained hilar sections of female apoE3-KI (***E***), female apoE4-KI (***F***), male apoE3-KI (***G***), and male apoE4-KI (***H***) mice at 16 months of age. ***I, J***, Escape latency in hidden platform days 1−5 correlated inversely with the number of somatostatin-positive hilar interneurons in female apoE4-KI mice (*I*, n = 12) but not female apoE3-KI mice (***J***, n = 10) at 16 months of age [28].

To determine the effects of sex, aging, and apoE4 on subpopulations of hilar GABAergic interneurons, we examined interneurons that were positive for somatostatin in female and male apoE-KI mice. At 1 month of age, female apoE3-KI mice exhibited 40% more somatostatin-positive interneurons than their male counterparts (Fig. 2*E, G, R*), although this sex difference was negligible in apoE4-KI mice (Fig. 2*F, H, R*). We further observed a greater age-dependent reduction in somatostatin-positive hilar interneurons in female apoE4-KI mice compared to age-matched female apoE3-KI mice (ANOVA: age, *F*_(4,54)_ = 4.756, *p*<0.01; apoE genotype, *F*_(1,54)_ = 23.85, *p*<0.005; interaction, *F*_(4,54)_ = 1.489, *p*>0.05) (Fig. 5*A*). This difference was first observed at 6 months of age (*post-hoc* Bonferroni; *p*<0.05) and persisted through 12 months (*post-hoc* Bonferroni; *p*<0.05) and 16 months (*post-hoc* Bonferroni; *p*<0.005) of age (Fig. 5*A, E, F*). Again, loss of these interneurons correlated with the observed spatial learning deficits in female apoE4-KI mice at 16 months of age (R^2^ = 0.521, *p* = 0.0085) (Fig. 5*J*), but not in female apoE3-KI mice (R^2^ = 0.026, *p* = 0.5821) (Fig. 5*I*). In contrast, male apoE-KI mice did not demonstrate any effect of apoE genotype on somatostatin-positive hilar interneuron levels (*post-hoc* Bonferroni; apoE genotype, *p*>0.05 for all ages examined), although we observed a clear age-dependent increase in number (ANOVA: age, *F*_(4,51)_ = 10.22, *p*<0.005; apoE genotype, *F*_(1,51)_ = 2.313, *p*>0.05; interaction, *F*_(4,51)_ = 0.1290, *p*>0.05) (Fig. 5*B, G, H*). Furthermore, there was no correlation between spatial learning ability and the number of somatostatin-positive hilar interneurons in male apoE-KI mice at 16 months of age (R^2^ = 0.0401, *p*>0.05 for male apoE3-KI mice; R^2^ = 0.0441, *p*>0.05 for male apoE4-KI mice). Sex had no effect among apoE3-KI mice with aging (ANOVA: age, *F*_(4,50)_ = 2.689, *p*<0.05; sex, *F*_(1,50)_ = 1.233, *p*>0.05; interaction, *F*_(4,50)_ = 7.321, *p*<0.001), although the number of somatostatin-positive interneurons was greater in 16-month old male apoE3-KI mice compared to age-matched female mice (*post-hoc* Bonferroni; *p*<0.05) (Fig. 5*C*). In contrast, apoE4-KI mice with aging demonstrated sex-dependent differences in hilar somatostatin-positive interneurons (ANOVA: age, *F*_(4,55)_ = 1.060, *p*>0.05; sex, *F*_(1,55)_ = 17.99, *p*<0.001; interaction, *F*_(4,55)_ = 7.461, *p*<0.001) (Fig. 5*D*). Age-related increases of this subtype of interneuron in male apoE4-KI mice led to greater numbers of somatostatin-positive interneurons compared to age-matched female apoE4-KI mice at age 12 months (*post-hoc* Bonferroni; *p*<0.001) and 16 months (*post-hoc* Bonferroni; *p*<0.001) (Fig. 5*D*). Similar to female apoE-KI mice, female wild-type mice exhibited an age-dependent decrease in hilar somatostatin-positive interneurons (ANOVA: age, *F*_(3,47)_ = 5.214, *p*<0.01) (Fig. 4*B*). By 16 months of age, female wild-type mice had significantly less somatostatin-positive hilar interneurons than their male counterparts (*post-hoc* Bonferroni; *p*<0.01) (Fig. 4*B*). Thus, apoE4 causes an age-dependent impairment of GAD67-positive and somatostatin-positive hilar GABAergic interneurons only in female apoE-KI mice, which correlates with the observed spatial learning deficits.

**Figure 6:**
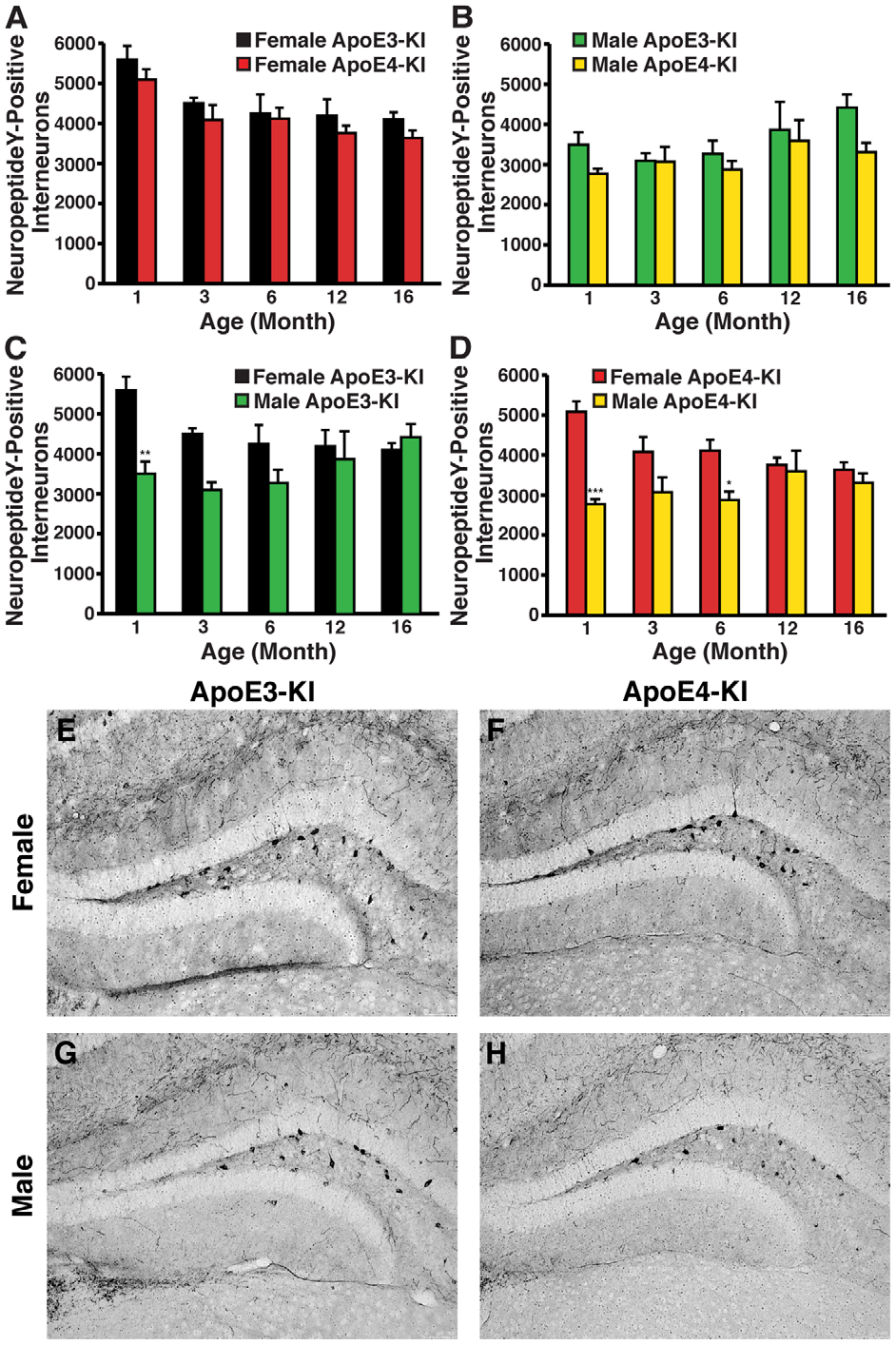
Age- and sex-dependent effects on numbers of neuropeptide Y-immunopositive hilar GABAergic interneurons in apoE-KI mice. ***A, B***, Histograms showing the total number of hilar GABAergic interneurons immunopositive for neuropeptide Y in female (***A***) or male (***B***) apoE-KI mice at the identified ages. Female and male apoE-KI mice show that NPY-positive interneuron levels are similarly apoE genotype-independent, but are age-dependent in opposite directions, whereby NPY-positive interneurons decrease and increase with age in females and males, respectively. There was no interaction between the two variables (by 2-way ANOVA) for either sex. ***C, D*** Quantification of NPY-positive hilar interneurons in female and male apoE3-KI (***C***) and apoE4-KI (***D***) mice at 1, 3, 6, 12, and 16 months of age (n = 6−12 mice per group). Both genotypes show age-independent, but sex-dependent, effects on NPY-positive interneurons by 2-way ANOVA. *p<0.05; **p<0.01; ***p<0.001 in male apoE-KI mice compared to female apoE-KI mice at the same age (*post-hoc* Bonferroni test). ***E–H***, Representative images (200x) of neuropeptide Y–immunostained sections of the hilus of female apoE3-KI (***E***), female apoE4-KI (***F***), male apoE3-KI (***G***), and male apoE4-KI (***H***) mice at 16 months of age.

We next assessed whether other subpopulations of hilar GABAergic interneurons were similarly affected by apoE4 expression in aged female mice. We first looked at the NPY-positive interneurons because its potential role in cognition is supported by a finding that targeted increase of NPY expression in the hippocampus improves memory performance [43]. Consistent with our GAD67 and somatostatin data, 1-month-old female apoE-KI mice showed a 50–80% increase in NPY-expressing hilar interneurons compared to male apoE-KI mice, regardless of apoE isoform (Fig. 2*I–L, S*). Temporal analysis of NPY-positive hilar interneurons in female apoE-KI mice revealed an overall age-dependent decrease in their numbers (ANOVA: age, *F*_(4,52)_ = 8.097, *p*<0.05), but no difference between apoE3-KI and apoE4-KI mice through to 16 months of age (*post-hoc* Bonferroni; apoE genotype, *p*>0.05 for all ages examined) (Fig. 6*A, E, F*). Male apoE-KI mice also showed no apoE genotype–dependent difference in the number of NPY-positive hilar interneurons with respect to age (*post hoc* Bonferroni; apoE genotype, *p*>0.05 for all ages examined) (Fig. 6*B, G, H*). A modest but significant age-dependent increase in NPY-positive hilar interneurons was present in male apoE-KI mice (ANOVA: age, *F*_(4,51)_ = 2.609, *p*<0.05) (Fig. 6*B*). No correlation between NPY-positive hilar interneurons and learning ability was apparent for either female or male apoE-KI mice at 16 months of age (females, R^2^ = 0.0873 for apoE3-KI mice, R^2^ = 0.0067 for apoE4-KI mice, *p*>0.05; males, R^2^ = 0.2895 for apoE3-KI mice, R^2^ = 5E-05 for apoE4-KI mice, *p*>0.05). Sex-dependent and age-independent changes were found in apoE3-KI mice (ANOVA: age, *F*_(4,50)_ = 1.606, *p*>0.05; sex, *F*_(1,50)_ = 14.11, *p*<0.001; interaction, *F*_(4,50)_ = 3.266, *p*<0.05) (Fig. 6*C*) and apoE4-KI mice (ANOVA: age, *F*_(4,55)_ = 0.8572, *p*>0.05; sex, *F*_(1,55)_ = 30.59, *p*<0.001; interaction, *F*_(4,55)_ = 4.608, *p*<0.01) (Fig. 6*D*). Female wild-type mice had a slight but significant age-dependent decrease in NPY-positive hilar interneurons (ANOVA: age, *F*_(3,47)_ = 3.031, *p*<0.05) with no significant difference compared to male wild-type mice at all ages analyzed (*post-hoc* Bonferroni; *p*>0.05) (Fig. 4*C*).

**Figure 7:**
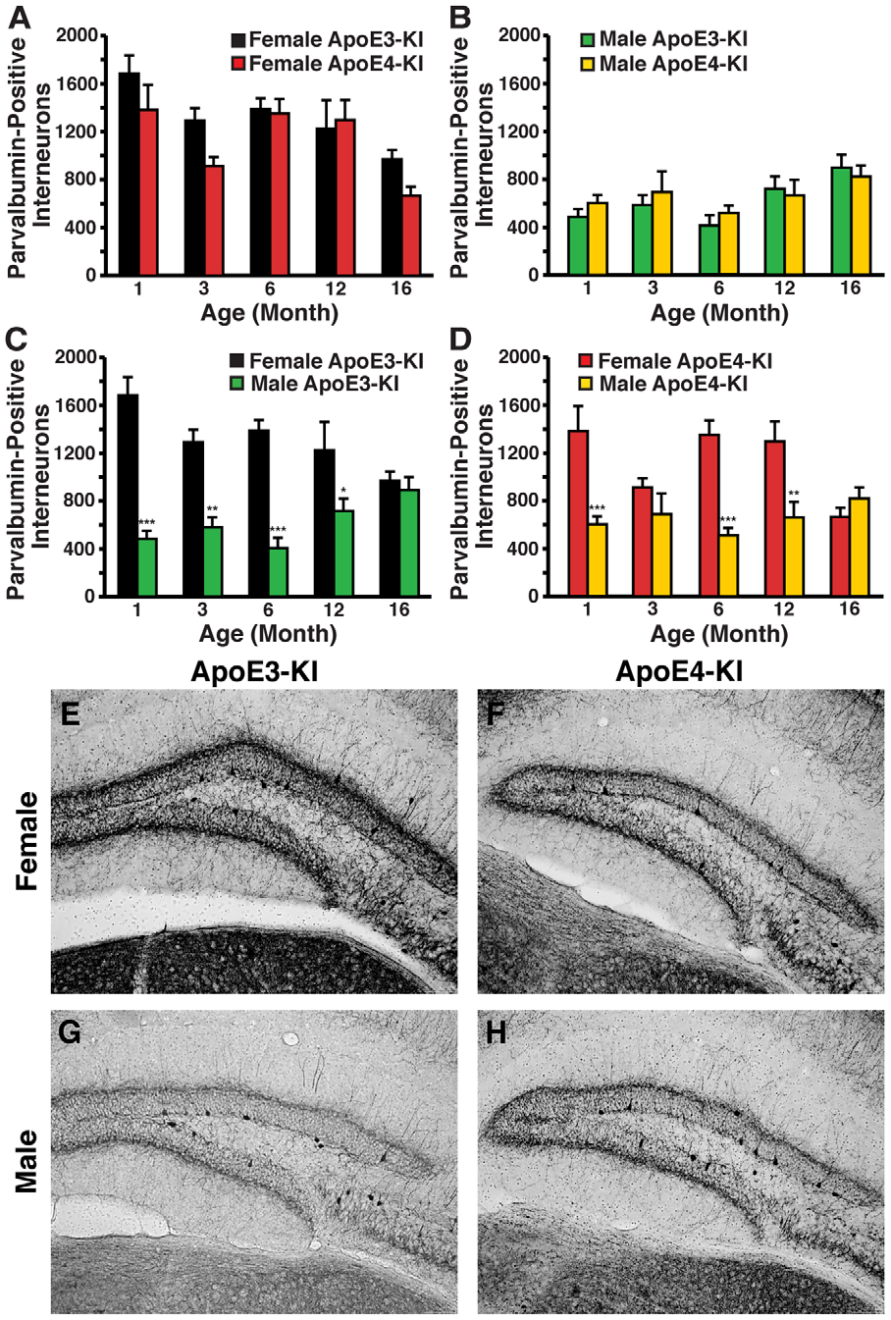
Age- and sex-dependent effects on numbers of parvalbumin-immunopositive hilar GABAergic interneurons in apoE-KI mice. ***A, B***, Histograms showing the total number of hilar GABAergic interneurons immunopositive for parvalbumin (PV) in female (***A***) or male (***B***) apoE-KI mice at the identified ages. Female apoE-KI mice show apoE genotype-independent and age-dependent effects, but no interaction between the two variables by 2-way ANOVA. Male apoE-KI mice show an age-dependent effect but no apoE genotype-dependent effect. There was no interaction between the two variables by 2-way ANOVA. ***C, D***, Quantification of parvalbumin-positive hilar interneurons in female and male apoE3-KI (***C***) and apoE4-KI (***D***) mice at 1, 3, 6, 12, and 16 months of age (n = 6−12 mice per group). Both apoE3-KI and apoE4-KI mice show an effect of sex on parvalbumin interneuron numbers. An interaction between age and sex was present for both apoE genotypes (2-way ANOVA). *p<0.05; **p<0.01; ***p<0.001 in male apoE-KI mice compared to female apoE-KI mice at the same age (*post-hoc* Bonferroni test). ***E–H***, Representative images (200x) of hilar sections stained with anti-parvalbumin antibodies. Shown are female apoE3-KI (***E***), female apoE4-KI (***F***), male apoE3-KI (***G***), and male apoE4-KI (***H***) mice at 16 months of age.

As parvalbumin-positive interneurons in the hippocampus are essential for spatial working memory [44], we also examined whether the numbers of parvalbumin-positive GABAergic interneurons in the hilus of the dentate gyrus were changed in apoE-KI mice as a function of age, sex, or apoE genotype. We found dramatically increased numbers of basal parvalbumin-positive hilar interneurons in 1-month-old female apoE-KI mice compared to male apoE-KI mice, where the females expressed approximately 2–4-fold more parvalbumin-positive interneurons than the males (Fig. 2*M–P, T*). Similar to the NPY findings, female apoE-KI mice demonstrated an age-dependent decrease in the number of parvalbumin-positive hilar interneurons (ANOVA; age, *F*_(4,52)_ = 8.849, *p*<0.005) that was most prevalent at 16 months of age (Fig. 7*A*). However, apoE isoform had no effect on this trend at any age (*post-hoc* Bonferroni; apoE genotype, *p*>0.05 for all ages examined). Male apoE-KI mice exhibited an increase in the number of parvalbumin-positive hilar interneurons that was age-dependent (ANOVA: age, *F*_(4,51)_ = 4.291, *p*<0.01) but independent of apoE isoform (*post-hoc* Bonferroni; apoE genotype, *p*>0.05 for all ages examined) (Fig. 7*B*). Interestingly, at 16 months of age, we observed very distinct differences in the morphology and projection patterns of parvalbumin-positive interneurons between the sexes. In female apoE-KI mice, the parvalbumin-positive interneurons within the hilus exhibited extensive processes that stretched to the molecular layer of the hippocampus; in contrast, these processes were nearly non-existent in their male counterparts (Fig. 7*E–H*) (*t*-test; axon quantitation: *p*<0.01 female versus male apoE3-KI mice; *p*<0.001 female versus male apoE4-KI mice) (Fig. S2 *B*). Learning ability was not correlated with the number of parvalbumin-positive interneurons in the hilus for either male or female apoE-KI mice at 16 months of age (females, R^2^ = 0.0235 for apoE3-KI mice, R^2^ = 0.2361 for apoE4-KI mice, *p*>0.05; males, R^2^ = 0.3127 for apoE3-KI mice, R^2^ = 0.1557 for apoE4-KI mice, *p*>0.05), suggesting that these changes occur independently of AD-related pathogenesis. Among apoE3-KI mice, sex had an effect on parvalbumin-positive interneuron numbers with aging (ANOVA: age, *F*_(4,50)_ = 0.5341, *p*>0.05; sex, *F*_(1,50)_ = 62.68, *p*<0.001; interaction, *F*_(4,50)_ = 4.818, *p*<0.01) (Fig. 7*C*). In particular, male mice exhibited significantly less than age-matched female mice through 12 months of age (*post-hoc* Bonferroni; *p*<0.001 at 1 month, *p*<0.01 at 3 months, *p*<0.001 at 6 months, *p*<0.05 at 12 months) (Fig. 7*C*). Similarly, apoE4-KI mice demonstrated age-related changes in parvalbumin-positive interneurons that were mediated by sex (ANOVA: age, *F*_(4,55)_ = 1.460, *p*>0.05; sex, *F*_(1,55)_ = 32.44, *p*<0.001; interaction, *F*_(4,55)_ = 5.207, *p*<0.01), with male mice having significantly less than female mice (*post-hoc* Bonferroni; *p*<0.001 at 1 month, *p*<0.001 at 6 months, *p*<0.01 at 12 months) (Fig. 7*D*). No age-dependent effects on parvalbumin-positive hilar interneurons were detected among female wild-type mice (ANOVA: age, *F*_(3,47)_ = 2.199, *p*>0.05), and total numbers were comparable to their male counterparts at all ages analyzed (*post-hoc* Bonferroni; *p*>0.05) (Fig. 4*D*). Thus, apoE4 has no effect on hilar NPY- or parvalbumin-positive interneuron profiles.

### The balance in inhibitory and excitatory interneurons in the hilus of apoE-KI mice is dependent on age, sex, and apoE isoform

**Figure 8:**
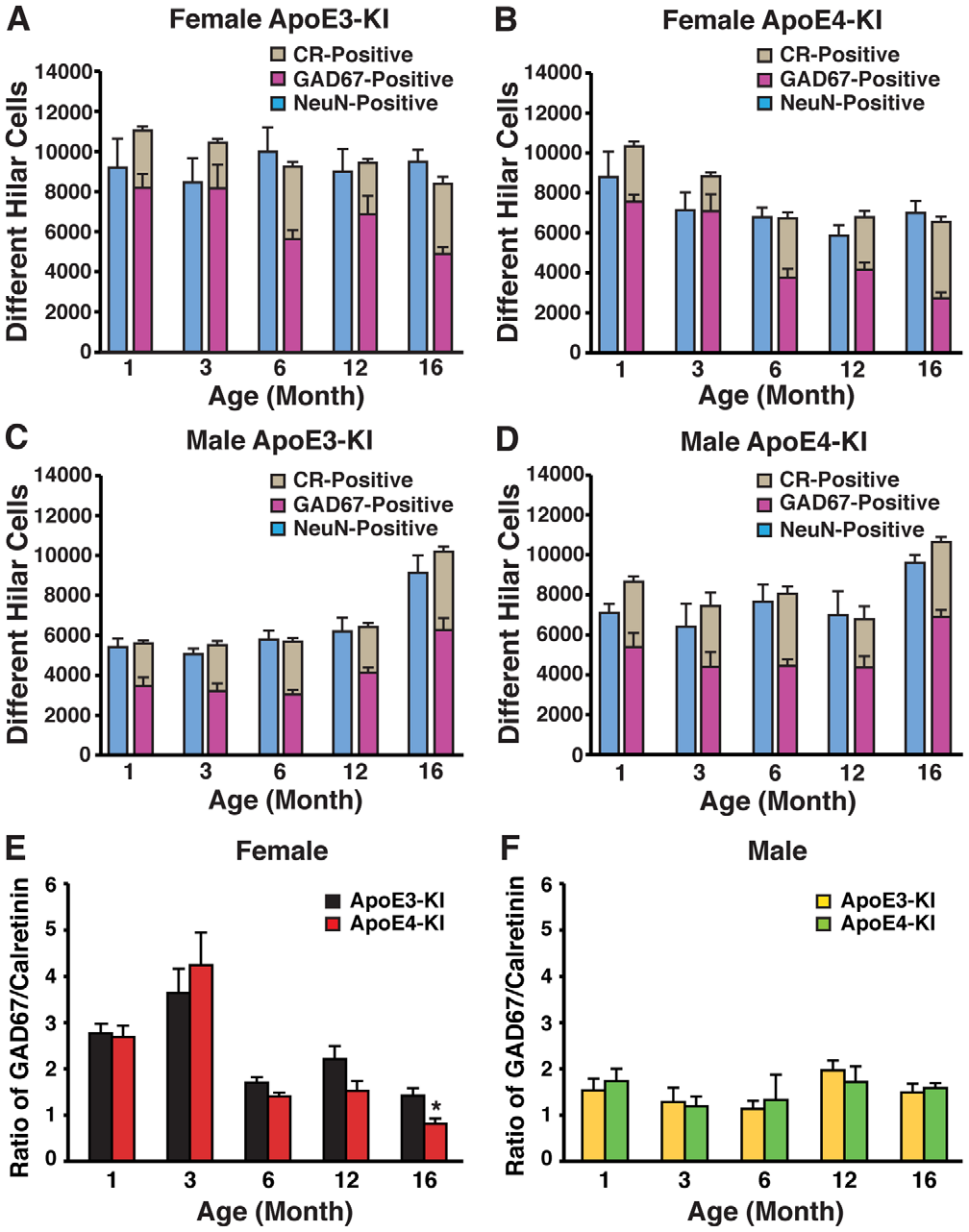
The balance between inhibitory and excitatory hilar interneurons depends on age, sex, and apoE isoform. ***A–D***, Histograms showing the number of hilar neurons positive for GAD67 (purple), calretinin (grey), or NeuN (blue) in female (***A, B***) and male (***C, D***) apoE3-KI (***A, C***) or apoE4-KI (***B, D***) mice at 1, 3, 6, 12, and 16 months of age (n = 6−12 mice per group). ***E-F***, Ratios of GAD67-positive interneurons to calretinin-positive mossy cells in the hilus of the dentate gyrus of female (***E***) and male (***F***) apoE-KI mice at 1, 3, 6, 12, and 16 months of age (n = 6−12 mice per group). *p<0.05 (*t*-test).

We next determined whether the reduction in the levels of GABAergic interneurons is caused by increased neuronal death or by loss of protein expression without cell death. To address this issue, we capitalized on evidence that neurons in the hilus are primarily inhibitory GABAergic interneurons or excitatory mossy cells [45], [46]. We performed immunostaining experiments that labeled all neurons (by NeuN), as well as inhibitory GABAergic interneurons (labeled by GAD67) and excitatory mossy cells (labeled by calretinin), and then quantified the numbers of neurons present in the hilus to identify whether the relative sum of GAD67-positive and calretinin-positive interneurons matched the relative total number of neurons in the same area. In the absence of immunoreactivity loss, the sum of GAD67-positive and calretinin-positive hilar neurons should account for the total number of NeuN-positive cells. Alternatively, reduced GAD67 immunoreactivity would reveal a disparity between the number of hilar NeuN-positive cells and the sum of GAD67-positive and calretinin-positive cells. In female apoE3-KI mice, there was no significant change in the number of hilar NeuN-positive cells with increasing age (Fig. 8*A*). Accordingly, the sum of GAD67-positive and calretinin-positive hilar cells showed little variation with age and approximately matched the number of NeuN-positive cells (Fig. 8*A*). In contrast, female apoE4-KI mice exhibited an age-dependent decrease in total NeuN-positive cells that corresponded with a decrease in the sum of GAD67-positive and calretinin-positive interneurons (Fig. 8*B*). Importantly, there were no significant changes in calretinin-positive cells with aging (Fig. 8*A, B*). This suggests that changes in the profiles of hilar GABAergic interneurons in female apoE4-KI mice were due to interneuron loss rather than reduced protein expression, and that the loss of GABAergic interneurons with age was exacerbated in female mice carrying the human apoE4 allele.

Interestingly, male apoE3-KI mice had an age-dependent overall increase in the number of hilar NeuN-positive cells, owing to increases in both GAD67- and calretinin-positive cell numbers (Fig. 8*C*). This result suggests that in male apoE3-KI mice, the increase in GAD67-positive interneurons with advancing age might involve GABAergic interneurogenesis. There was also a trend toward age-dependent increases in hilar neuronal cells in male apoE4-KI mice although it did not reach statistical significance (Fig. 8*D*). Similar to the other groups, the sum of GAD67-positive and calretinin-positive hilar interneurons accounted for the number of hilar NeuN-positive cells (Fig. 8*D*). These results suggest that GAD67 immunoreactivity is indeed an accurate reflection of the number and distribution of GABAergic interneurons for both male and female apoE-KI mice. Thus, apoE4 exacerbates age-dependent GABAergic interneuron loss in female apoE-KI mice, whereas male apoE-KI mice show an age-dependent, but apoE isoform-independent, increase in the levels of GABAergic interneurons.

Finally, we examined the ratio of GAD67-positive inhibitory interneurons to calretinin-positive excitatory mossy cells in the hilus of female and male apoE-KI mice. Male apoE-KI mice consistently showed, independent of apoE genotype and age, a ratio approximating 1.5, indicating the presence of about 50% more inhibitory GABAergic interneurons than excitatory mossy cells (Fig. 8*F*). In contrast, female apoE-KI mice exhibited an age-dependent decrease in the ratio of GABAergic interneurons to mossy cells that was exacerbated by the presence of apoE4, especially at 16 months of age (*t*-test, *p*<0.05) (Fig. 8*E*). At younger ages, female apoE3-KI and apoE4-KI mice had 2.5 to 3.5-fold more GABAergic interneurons compared to mossy cells (Fig. 8*E*). However, at 16 months of age, the number of GAD67-positive interneurons was approximately equivalent to the number of calretinin-positive cells in female apoE3-KI mice (Fig. 8*E*). In contrast, female apoE4-KI mice at this age demonstrated a ratio of less than 1, indicating the presence of fewer inhibitory GABAergic interneurons than excitatory mossy cells (Fig. 8*E*). These findings suggest that the balance of inhibitory and excitatory interneurons in the hilus of apoE-KI mice is age- and sex-dependent, with apoE4 compromising this balance in aged female mice only.

### Aged male apoE-KI mice, but not aged female apoE-KI mice, exhibit hilar GABAergic interneurogenesis

**Figure 9:**
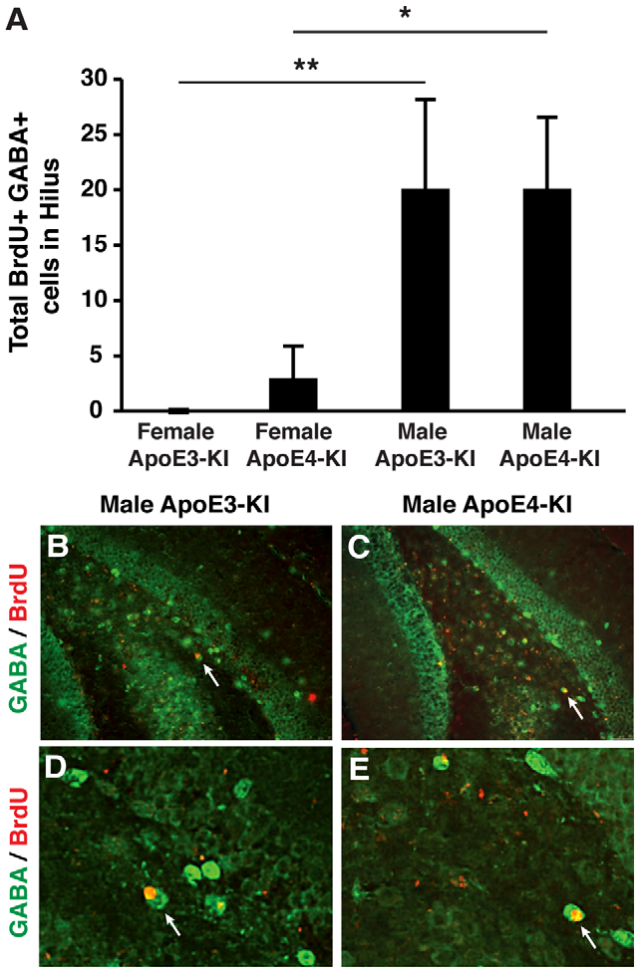
GABAergic interneurogenesis in the hilus is sex-dependent in aged apoE-KI mice. ***A***, Total numbers of BrdU and GABA double-positive cells in the hilus of female and male apoE-KI mice at 16 months of age were determined 1 month after BrdU injection. Values are mean ± SE (n = 6−8 mice per group). *p<0.05 in 16-month-old male apoE4-KI mice compared to their female counterparts (*post-hoc* Bonferroni test); **p<0.01 in older male apoE3-KI mice compared to their female counterparts at the same age (*post-hoc* Bonferroni test). ***B, D***, Images of the hilus of 16-month-old male apoE3-KI mice stained with anti-BrdU (red) and anti-GABA (green) collected 1 month after BrdU injection (magnification: ***B***, 100x; ***D***, 200x). ***C, E***, Images of the hilus of 16-month-old male apoE4-KI mice stained with anti-BrdU (red) and anti-GABA (green) collected 1 month after BrdU injection (magnification: ***C***, 100x; ***E***, 200x).

Because apoE-KI mice demonstrated an age-dependent and sex-specific increase in numbers of hilar GABAergic interneurons, we investigated the possibility of GABAergic interneurogenesis in the hilus of aged male brains. Male and female apoE-KI mice were sacrificed 4 weeks after BrdU injection (at 16 months of age), and their brains were sectioned and processed for BrdU and GABA double immunolabeling in the hilus. We found little to no BrdU/GABA double-labeled cells in the hilus of 16-month-old female apoE-KI mice, whereas male apoE-KI mice at the same age had significant numbers of BrdU and GABA double-positive cells in the hilus (Fig. 9*A–E*). These results suggest that the increase in GABAergic interneurons in the hilus of aged male mice was due, in part, to interneurogenesis.

### ApoE4-mediated impairment of GABAergic interneurons in female apoE-KI mice occurs predominantly in the hilus

To test whether the GABAergic interneuron impairments in female apoE4-KI mice were specific to the hilus, we examined whether such changes also occurred–with respect to age, sex and apoE genotype–in several areas involved in the hippocampal circuitry. Notably, we inspected the adjoining entorhinal cortex, which sends axons along the perforant pathway to the dentate gyrus; the CA3, which receives input from the dentate granule cells; and the CA1, which receives input from CA3 pyramidal cells. Interneurons in the auditory cortex were also quantified as an extra-hippocampal control.

We first investigated the entorhinal cortex, a region in which alterations have been observed in association with several disorders of the human brain, importantly AD, temporal lobe epilepsy and schizophrenia. Female apoE-KI mice showed an age-dependent decline in the number of interneurons positive for GAD67 (ANOVA: age, *F*_(4,50)_ = 26.97, *p*<0.001) (Fig. S3 *A*), owing to universal age-related decreases in interneurons positive for somatostatin (ANOVA: age, *F*_(4,50)_ = 20.25, *p*<0.001) (Fig. S3 *C*), NPY (ANOVA: age, *F*_(4,52)_ = 9.910, *p*<0.001) (Fig. S3 *E*), and parvalbumin (ANOVA: age, *F*_(4,52)_ = 11.87, *p*<0.001) (Fig. S3 *G*). Male apoE-KI mice similarly exhibited a collective decrease of GAD67-positive (ANOVA: age, *F*_(4,51)_ = 9.360, *p*<0.001) (Fig. S3 *B*), somatostatin-positive (ANOVA: age, *F*_(4,51)_ = 10.17, *p*<0.001) (Fig. S3 *D*), NPY-positive (ANOVA: age, *F*_(4,51)_ = 11.43, *p*<0.001) (Fig. S3 *F*), and parvalbumin-positive (ANOVA: age, *F*_(4,51)_ = 11.19, *p*<0.001) (Fig. S3 *H*) interneurons with advancing age. ApoE isoform had no effect in the entorhinal cortex of female and male apoE-KI mice, with one exception where 1-month old male apoE3-KI mice had more parvalbumin-positive interneurons than their apoE4-KI counterparts (Table 1 *and* Fig. S3 *H*). Evidently, numbers of GABAergic interneurons in the entorhinal cortex decrease as a natural function of age in females and males, with minimal apoE genotype effect at any age.

**Table 1:** Summary of the effects of apoE genotype on GABAergic interneuron numbers in the entorhinal cortex, the CA1 and CA3 subregions of the hippocampus, and the auditory cortex in female and male apoE-KI mice.

	GAD67		Somatostatin		NeuropeptideY		Parvalbumin	
Area	Female	Male	Female	Male	Female	Male	Female	Male
Entorhinal Cortex	No effect	No effect	No effect	No effect	No effect	No effect	No effect	Yes*
CA3	No effect	No effect	No effect	No effect	No effect	No effect	No effect	No effect
CA1	No effect	No effect	No effect	No effect	No effect	No effect	Yes**	No effect
Auditory Cortex	No effect	No effect	No effect	No effect	No effect	No effect	No effect	No effect

* Male apoE3-KI > male apoE4-KI at 1 month of age (*post-hoc* Bonferroni; *p*<0.01).

** Female apoE3-KI > female apoE4-KI at 1 month of age (*post-hoc* Bonferroni; *p*<0.01).

The CA3 subregion has also been implicated as a vulnerable area in the course of AD pathogenesis. In this area, we found that female apoE-KI mice showed an age-related decline in NPY-positive (ANOVA: age, *F*_(4,50)_ = 2.676, *p*<0.05) (Fig. S4 *E*) and parvalbumin-positive (ANOVA: age, *F*_(4,52)_ = 3.302, *p*<0.05) interneurons (Fig. S4 *G*), but no age-dependent effects on GAD67-positive (ANOVA: age, *F*_(4,50)_ = 0.5183, *p*>0.05) (Fig. S4 *A*) and somatostatin-positive (ANOVA: age, *F*_(4,50)_ = 1.345, *p*>0.05) interneurons (Fig. S4 *C*). Conversely, an age-related increase in cells positive for GAD67 (ANOVA: age, *F*_(4,51)_ = 17.28, *p*<0.001) (Fig. S4 *B*) was noted among male apoE-KI mice, likely due to increases in somatostatin-positive (ANOVA: age, *F*_(4,51)_ = 9.001, *p*<0.001) (Fig. S4 *D*) and parvalbumin-positive (ANOVA: age, *F*_(4,51)_ = 9.592, *p*<0.001) (Fig. S4 *H*) interneurons with advancing age. No age effect on NPY-positive interneurons in CA3 subfield was found (ANOVA: age, *F*_(4,51)_ = 1.899, *p*>0.05) (Fig. S4 *F*). GABAergic interneuron numbers in CA3 were not modified by apoE genotype for either female or male apoE-KI mice (*post-hoc* Bonferroni; apoE genotype, *p*>0.05 for all ages examined) (Table 1).

We further investigated the effect of apoE4 and sex on age-related changes in CA1 inhibitory interneurons, as neurons in the CA1 field are heavily damaged in AD brains compared to other hippocampal subregions. Similar to the pattern seen in the CA3 subfield, an age-dependent decrease in NPY-positive (ANOVA: age, *F*_(4,50)_ = 3.242, *p*<0.05) (Fig. S5 *E*) and parvalbumin-positive (ANOVA: age, *F*_(4,52)_ = 10.08, *p*<0.001) (Fig. S5 *G*) interneurons, contributing to an age-dependent reduction in GAD67-positive interneurons (ANOVA: age, *F*_(4,50)_ = 3.618, *p*<0.05) (Fig. S5 *A*), was observed in the CA1 of female apoE-KI mice. Age had no effect on somatostatin-positive interneurons in this region (ANOVA: age, *F*_(4,50)_ = 1.055, *p*>0.05) (Fig. S5 *C*). A general increase in numbers of GABAergic interneurons with advancing age was noted among male apoE-KI mice. This was true for cells positive for GAD67 (ANOVA: age, *F*_(4,51)_ = 11.42, *p*<0.001) (Fig. S5 *B*), somatostatin (ANOVA: age, *F*_(4,51)_ = 7.670, *p*<0.001) (Fig. S5 *D*), NPY (ANOVA: age, *F*_(4,51)_ = 2.697, *p*<0.05) (Fig. S5 *F*), and parvalbumin (ANOVA: age, *F*_(4,51)_ = 8.958, *p*<0.001) (Fig. S5 *H*). With one exception, apoE genotype had no effect on GABAergic interneurons in the CA1 region among female and male apoE-KI mice (*post-hoc* Bonferroni; apoE genotype, *p*>0.05 for all ages examined, except **p<0.01 in female apoE4-KI mice compared to female apoE3-KI mice at 1 month of age) (Table 1). Thus, apoE genotype-associated modifications of GABAergic interneurons within the hippocampal formation are largely specific to the hilus. Interestingly, male apoE-KI mice reveal an age-dependent increase in CA3 and CA1 GABAergic interneurons consistent with the pattern found in the hilus, whereas female apoE-KI mice demonstrate an age-dependent decrease.

Sex- and apoE genotype–related GABAergic interneuron alterations in the auditory cortex were examined as an extra-hippocampal control, as this area has no known role in cognitive deficits associated with AD. Female apoE-KI mice had a ubiquitous age-related decline in interneurons positive for somatostatin (ANOVA: age, *F*_(4,50)_ = 3.556, *p*<0.05) (Fig. S6 *C*), NPY (ANOVA: age, *F*_(4,52)_ = 10.67, *p*<0.001) (Fig. S6 *E*), and parvalbumin (ANOVA: age, *F*_(4,52)_ = 12.49, *p*<0.001) (Fig. S6 *G*) in this region, contributing to an overall age-dependent decrease in GAD67-positive interneurons (ANOVA: age, *F*_(4,50)_ = 12.60, *p*<0.001) (Fig. S6 *A*). Male apoE-KI mice had age-dependent increases in GAD67-positive interneurons in the auditory cortex (ANOVA: age, *F*_(4,51)_ = 6.048, *p*<0.001) (Fig. S6 *B*), owing predominantly to a parallel age-related rise in parvalbumin-positive interneuron numbers (ANOVA: age, *F*_(4,51)_ = 2.844, *p*<0.05) (Fig. S6 *H*). The levels of somatostatin-positive (ANOVA: age, *F*_(4,51)_ = 6.687, *p*<0.001) (Fig. S6 *D*) and NPY-positive interneurons (ANOVA: age, *F*_(4,50)_ = 9.005, *p*<0.001) (Fig. S6 *F*) decreased with age. ApoE genotype did not modulate the numbers of GABAergic interneurons in the auditory cortex (*post-hoc* Bonferroni; apoE genotype, *p*>0.05 for all ages examined in female and male apoE-KI mice) (Table 1). Taken together, these findings indicate prominent sex differences in age-related changes of inhibitory neurons, and moreover, a specific effect of apoE4 on hilar GABAergic interneurons.

## Discussion

Although many epidemiological and clinical studies have identified sex differences in AD susceptibility among apoE4 carriers, the mechanism responsible for this discrepancy is unknown. Unexpectedly, our data revealed that independent of apoE genotype, male apoE-KI mice had an age-dependent increase in the number of hilar GABAergic interneurons while female apoE-KI mice demonstrated an age-dependent decrease, due in part to sex-specific GABAergic interneurogenesis in the hilus of aged male apoE-KI mice that is little or absent in aged female apoE-KI mice. We also demonstrate that the apoE4-induced impairments of spatial learning and memory are specific to female mice, consistent with human epidemiological AD findings. Compared to female apoE3-KI mice, female apoE4-KI mice had greater age-dependent decreases in select subpopulations of hilar GABAergic interneurons, which correlated with the presence of spatial learning and memory deficits. In contrast, male apoE-KI mice exhibited normal spatial learning and memory regardless of apoE genotype, and accordingly, showed apoE genotype-independent changes in hilar GABAergic interneurons with aging. Detailed analysis of the interneuron population in the hilus of male and female apoE-KI mice revealed that only female apoE-KI mice showed dramatic age-dependent alterations in the balance between inhibitory and excitatory interneurons that were exacerbated by apoE4 expression. Moreover, our data revealed that these apoE4-mediated interneuron changes occur only in the hilus. Our findings suggest that the discrepancies in sex susceptibility to developing AD may be attributable to inherent differences in hilar GABAergic interneuron levels, which is further modulated by apoE genotype.

The GABAergic system is critically involved in cognitive processes, particularly learning and memory [47], [48], and dysfunction of the GABAergic system may contribute to cognitive impairments such as those observed in AD [49]. Clinical studies have demonstrated a link between AD-related dementia and alterations in GABA or somatostatin levels in the brain and CSF [50], [51], [52], [53], [54] that are exacerbated by apoE4 [55]. A single nucleotide polymorphism of the somatostatin gene is associated with increased risk for AD in apoE4, but not apoE3, carriers [56], [57]. Furthermore, GABA levels in human CSF decrease with age [53]. Conversely, interventions that increase GABAergic interneuron expression or transmission have been shown to improve cognitive function. For example, increased GABA levels, resulting from knocked down expression of the GABA transporter-1 gene, are associated with enhanced spatial learning and memory [58], and pharmacological facilitation of somatostatinergic activity has been shown to ameliorate memory deficits [59]. Here, we reveal sex discrepancies in the GABAergic system of apoE-KI mice whereby GABAergic interneurons in female apoE4-KI are particularly vulnerable to age-related declines compared to their male counterparts, which is concurrent with cognitive deficits present specifically in female mice that carry the apoE4 gene.

The molecular mechanisms contributing to the age-, sex-, and apoE isoform-dependent GABAergic interneuron loss in the hilus of apoE-KI mice are still unclear, although the inherent discrepancies in the hippocampal GABAergic system observed in the present study may be explained, at least in part, by differences in the expression of sex hormones. Sex steroids are known to influence brain organization during development, which can subsequently affect cognitive behaviors in adulthood [60]. In the rodent hippocampus, androgen receptor (AR)-specific immunoreactivity and AR binding are concentrated in CA1 pyramidal cell nuclei [61], [62], and diffuse AR immunoreactivity is distributed in the mossy fiber pathway [63]. These findings implicate a regulatory role for androgens in hippocampal function, although the absence of AR expression in GABAergic interneuron nuclei [63] suggests that androgens may not act directly to modulate GABAergic interneuron function. Estrogens may also influence spatial learning and memory [64], [65], and, notably, nuclear estrogen receptors are expressed in GABA-producing hippocampal interneurons [66], [67], [68], [69], [70], suggesting that estrogens regulate the morphogenesis and/or activity of GABAergic interneurons. Indeed, acute or repeated administration of physiological doses of β-estradiol in ovariectomized female rodents results in the upregulation of NPY-positive GABAergic interneurons in the hilus [41], [71], leading to the functional augmentation of granule cell network inhibition [41]. In the hippocampus, estradiol increases mRNA transcript levels of GAD, the GABA synthesizing enzyme, in ovariectomized rats [72], while depletion of estrogen levels by ovariectomy decreases the number of GAD-expressing neurons [73]. Thus, it is likely that elevated levels of estrogen in younger female apoE-KI mice serve to maintain higher numbers of GABAergic interneurons, while age-dependent decreases in estrogen result in reduced GABAergic interneuron levels. In males, testosterone levels decrease as a natural consequence of aging [74], [75], [76]. However, estrogen levels in the brain, produced by the local aromatization of testosterone [77], increase with age in males [78] due to elevated levels and activity of aromatase [79], [80]. It is thus reasonable to speculate that in males, age-related increases in estrogen levels promote the observed increase in the levels of GABAergic interneurons.

The signaling mechanisms that translate hilar GABAergic interneuron impairments into cognitive dysfunction are undetermined, although tau may play a prominent role [81], [82]. Female transgenic mice that express low levels of a carboxyl-terminal-truncated fragment of apoE4 (apoE4(Δ272–299)) in neurons exhibit neurodegeneration, abnormally high levels of hyperphosphorylated tau in the brain, and cognitive dysfunction [42]. Importantly, these apoE4(Δ272–299) mice also demonstrate impairments of hilar GABAergic interneurons that correlated with spatial learning deficits [28]. Abolishing tau expression ameliorated the learning and memory impairments as well as the detrimental effects of apoE4(Δ272–299) on hilar GABAergic interneurons, suggesting that tau is a crucial downstream mediator in apoE4-related GABAergic interneuron impairment and AD pathogenesis [28].

The GABAergic system also plays an important role in the etiology of epilepsy. Epileptic seizures frequently occur in early-onset and advanced AD patients [83], [84], and seizures may be a reflection of pathological processes similar to or overlapping with those responsible for cognitive decline. Numerous studies have demonstrated alterations in the number and function of GABAergic interneurons in the cortex and hippocampus of animal models and patients with epilepsy [85], [86], [87], [88], [89], [90]. In the context of AD, hAPP transgenic mice–which overexpress human amyloid peptides, leading to AD-like symptoms–exhibit spontaneous nonconvulsive seizure activity in cortical and hippocampal networks that is associated with aberrant GABAergic sprouting in the dentate gyrus [91]. Interestingly, female mice are more susceptible to seizures triggered by pentylenetetrazol, a GABA antagonist, than male mice [92], suggesting that the likelihood of developing epilepsy could be influenced by sex.

Our analysis shows that aged female apoE4-KI mice have an altered balance of inhibitory GABAergic interneurons and excitatory mossy cells in the hilus compared to both female apoE3-KI mice and male apoE-KI mice of any apoE genotype. ApoE4 is associated with increased network excitability and stress-induced subclinical epileptiform activity [93]. Thus, it is possible that female apoE4-KI mice similarly have abnormal network excitability as a result of imbalanced inhibitory and excitatory activity between GABAergic interneurons and mossy cells, respectively. Accordingly, female apoE4-KI mice have increased seizure susceptibility compared to male apoE4-KI mice [94]. Our studies here reveal an inherent discrepancy in the hippocampal GABAergic system between male and female apoE-KI mice, which may form the basis for the links among apoE isoforms, sex, and AD. Clearly, understanding apoE-sex-based interactions in the context of advancing age is of fundamental importance, and can yield important insight that could advance treatment and prevention strategies for AD. Further investigations into the functional aspects of hilar GABAergic interneuron signaling and regulation, as well as its role in cognitive function, is therefore critical to the development of safe and effective AD therapies.

## Supporting Information

Figure S1 12-month-old female and male apoE-KI mice show normal spatial learning and memory. ***A***, 12-month-old male and female apoE3-KI or apoE4-KI mice (n = 10−13 mice per group) were tested in the Morris water maze. Points represent averages of daily trials. H, hidden platform day (2 trials/session, 2 sessions/day); H0, first trial on H1; V, visible platform day (2 trials/session, 2 sessions/day). Escape latency (*y*-axis) indicates time to reach the target. Male and female apoE-KI mice perform at a similar level independent of apoE genotype (repeated-measures ANOVA, *p*>0.05; *post-hoc* comparisons: apoE3-KI vs apoE4-KI, *p*>0.05 for both male and female). ***B***, Swim speed was not different among the various groups of mice. ***C, D***, Probe 1 trials of female (***C***, n = 11−13) and male (***D***, n = 10−12) apoE3-KI or apoE4-KI mice were performed 24 h after the last hidden day platform training. Percentage time spent in the target quadrant versus the time spent in any of the three non-target quadrants differed in all groups. ***E, F***, Probe 2 trials of female (***E***, n = 11−13) and male (***F***, n = 10−12) apoE3-KI and apoE4-KI mice were performed 72 h after the last hidden day platform training. Percentage time spend in the target quadrant versus the time spent in any of the three non-target quadrants differed in all groups. **p<0.01, ***p<0.001 (*t*-test).
(TIF)

Figure S2 Neuronal processes of hilar GABAergic interneurons differ by sex. ***A***, Quantification of GAD67 immunoreactivity (IR) in the hilus of 1-month-old male and female apoE-KI mice (n = 6 mice per group). Male apoE-KI mice show greater hilar GAD67-IR compared to their female counterparts. ***p<0.001 male apoE-3KI versus female apoE3-KI mice (*t*-test); ** *p*<0.01 male apoE4-KI versus female apoE4-KI mice (*t*-test). ***B***, Quantification of parvalbumin immunoreactivity (IR) in the processes extending from parvalbumin-positive interneurons in 16-month old male and female apoE-KI mice (n = 6−12 mice per group). Female apoE-KI mice have more extensive processes than male apoE-KI mice. **p<0.01 female versus male apoE3-KI mice (*t*-test); ***p<0.001 female versus male apoE4-KI mice (*t*-test).
(TIF)

Figure S3 GABAergic interneuronal profiles in the entorhinal cortex change as a function of age, sex and apoE genotype. ***A–H***, GABAergic interneurons in the entorhinal cortex positive for GAD67 (***A, B***), somatostatin (***C, D***), neuropeptide Y (***E, F***), and parvalbumin (***G, H***) in female (***A, C, E, G***) and male (***B, D, F, H***) apoE-KI mice at 1, 3, 6, 12, and 16 months of age (n = 6−12 mice per group). Results in histograms are presented as the total number of positive cells counted per brain.
(TIF)

Figure S4 GABAergic interneuronal profiles in the CA3 change as a function of age, sex and apoE genotype. ***A–H***, GABAergic interneurons in the CA3 positive for GAD67 (***A, B***), somatostatin (***C, D***), neuropeptide Y (***E, F***), and parvalbumin (***G, H***) in female (***A, C, E, G***) and male (***B, D, F, H***) apoE-KI mice at 1, 3, 6, 12, and 16 months of age (n = 6−12 mice per group). Results in histograms are presented as the total number of positive cells counted per brain.
(TIF)

Figure S5 GABAergic interneuronal profiles in the CA1 change as a function of age, sex and apoE genotype. ***A–H***, GABAergic interneurons in the CA1 positive for GAD67 (***A, B***), somatostatin (***C, D***), neuropeptide Y (***E, F***), and parvalbumin (***G, H***) in female (***A, C, E, G***) and male (***B, D, F, H***) apoE-KI mice at 1, 3, 6, 12, and 16 months of age (n = 6−12 mice per group). Results in histograms are presented as the total number of positive cells counted per brain. **p<0.01.
(TIF)

Figure S6 GABAergic interneurons in the auditory cortex change as a function of age, sex and apoE genotype. ***A–H***, GABAergic interneurons in the auditory cortex positive for GAD67 (***A, B***), somatostatin (***C, D***), neuropeptide Y (***E, F***), and parvalbumin (***G, H***) in female (***A, C, E, G***) and male (***B, D, F, H***) apoE-KI mice at 1, 3, 6, 12, and 16 months of age (n = 6−12 mice per group). Results in histograms are presented as the total number of positive cells counted per brain.
(TIF)
