# Supplementary figures and images for: Apolipoprotein E4 Causes Age- and Sex-Dependent Impairments of Hilar GABAergic Interneurons and Learning and Memory Deficits in Mice
Source: PLoS One. 2012 Dec 31;7(12):e53569. doi: 10.1371/journal.pone.0053569 (PMC3534053; doi:10.1371/journal.pone.0053569)

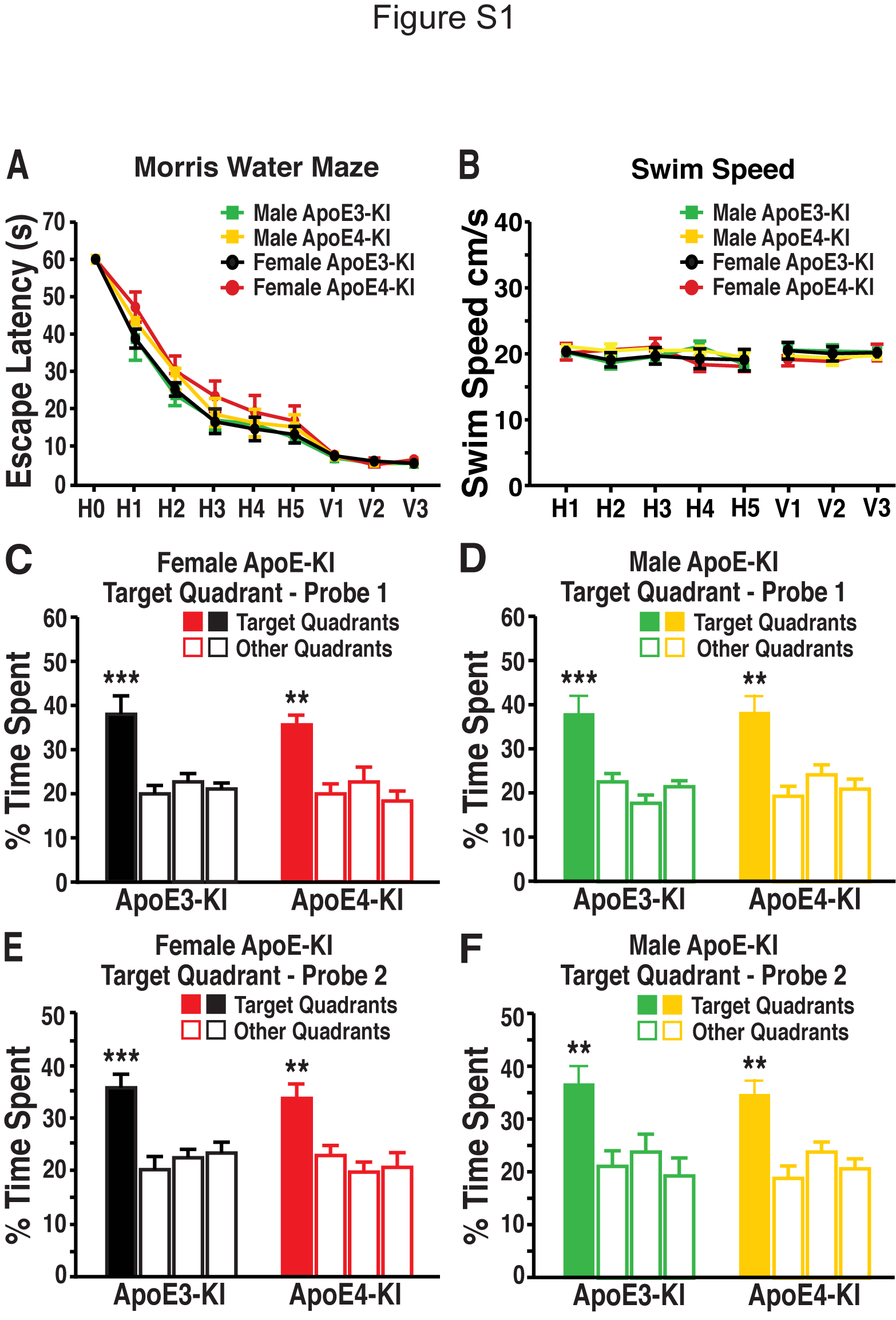

Supplement: Figure S1 — 12-month-old female and male apoE-KI mice show normal spatial learning and memory. A, 12-month-old male and female apoE3-KI or apoE4-KI mice (n = 10−13 mice per group) were tested in the Morris water maze. Points represent averages of daily trials. H, hidden platform day (2 trials/session, 2 sessions/day); H0, first trial on H1; V, visible platform day (2 trials/session, 2 sessions/day). Escape latency (y-axis) indicates time to reach the target. Male and female apoE-KI mice perform at a similar level independent of apoE genotype (repeated-measures ANOVA, p>0.05; post-hoc comparisons: apoE3-KI vs apoE4-KI, p>0.05 for both male and female). B, Swim speed was not different among the various groups of mice. C, D, Probe 1 trials of female (C, n = 11−13) and male (D, n = 10−12) apoE3-KI or apoE4-KI mice were performed 24 h after the last hidden day platform training. Percentage time spent in the target quadrant versus the time spent in any of the three non-target quadrants differed in all groups. E, F, Probe 2 trials of female (E, n = 11−13) and male (F, n = 10−12) apoE3-KI and apoE4-KI mice were performed 72 h after the last hidden day platform training. Percentage time spend in the target quadrant versus the time spent in any of the three non-target quadrants differed in all groups. **p<0.01, ***p<0.001 (t-test). (TIF) [file pone.0053569.s001.tif]

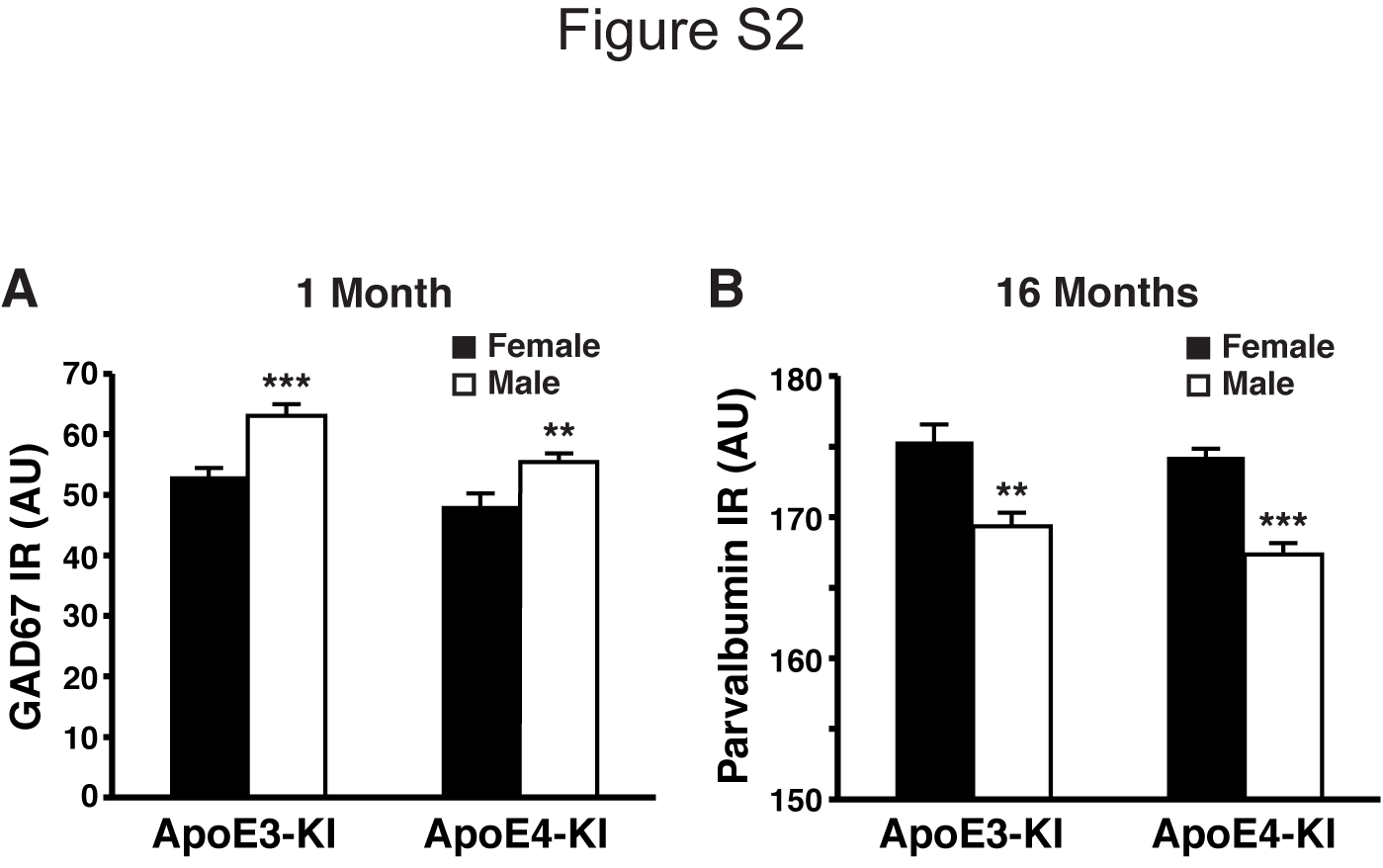

Supplement: Figure S2 — Neuronal processes of hilar GABAergic interneurons differ by sex. A, Quantification of GAD67 immunoreactivity (IR) in the hilus of 1-month-old male and female apoE-KI mice (n = 6 mice per group). Male apoE-KI mice show greater hilar GAD67-IR compared to their female counterparts. ***p<0.001 male apoE-3KI versus female apoE3-KI mice (t-test); ** p<0.01 male apoE4-KI versus female apoE4-KI mice (t-test). B, Quantification of parvalbumin immunoreactivity (IR) in the processes extending from parvalbumin-positive interneurons in 16-month old male and female apoE-KI mice (n = 6−12 mice per group). Female apoE-KI mice have more extensive processes than male apoE-KI mice. **p<0.01 female versus male apoE3-KI mice (t-test); ***p<0.001 female versus male apoE4-KI mice (t-test). (TIF) [file pone.0053569.s002.tif]

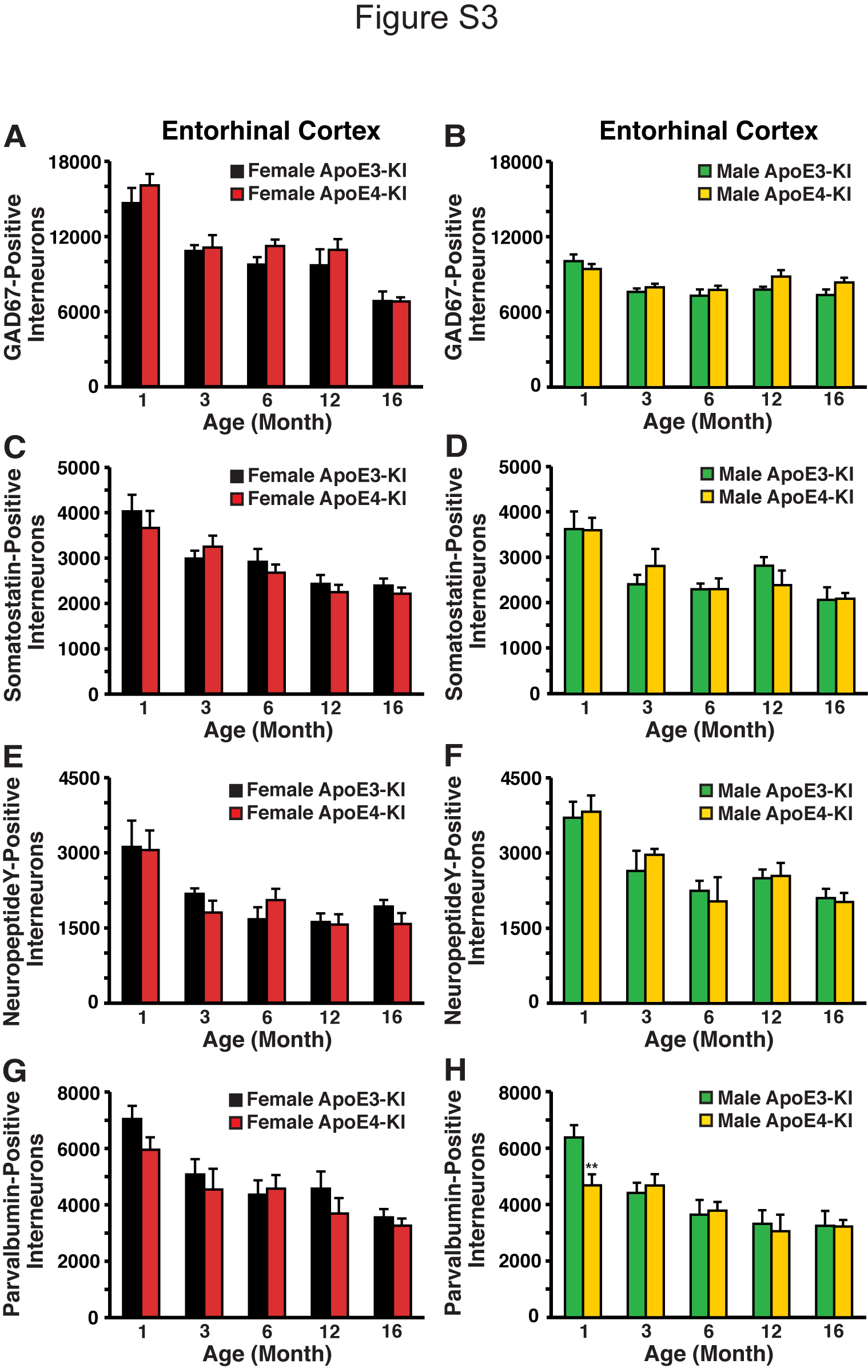

Supplement: Figure S3 — GABAergic interneuronal profiles in the entorhinal cortex change as a function of age, sex and apoE genotype. A–H, GABAergic interneurons in the entorhinal cortex positive for GAD67 (A, B), somatostatin (C, D), neuropeptide Y (E, F), and parvalbumin (G, H) in female (A, C, E, G) and male (B, D, F, H) apoE-KI mice at 1, 3, 6, 12, and 16 months of age (n = 6−12 mice per group). Results in histograms are presented as the total number of positive cells counted per brain. (TIF) [file pone.0053569.s003.tif]

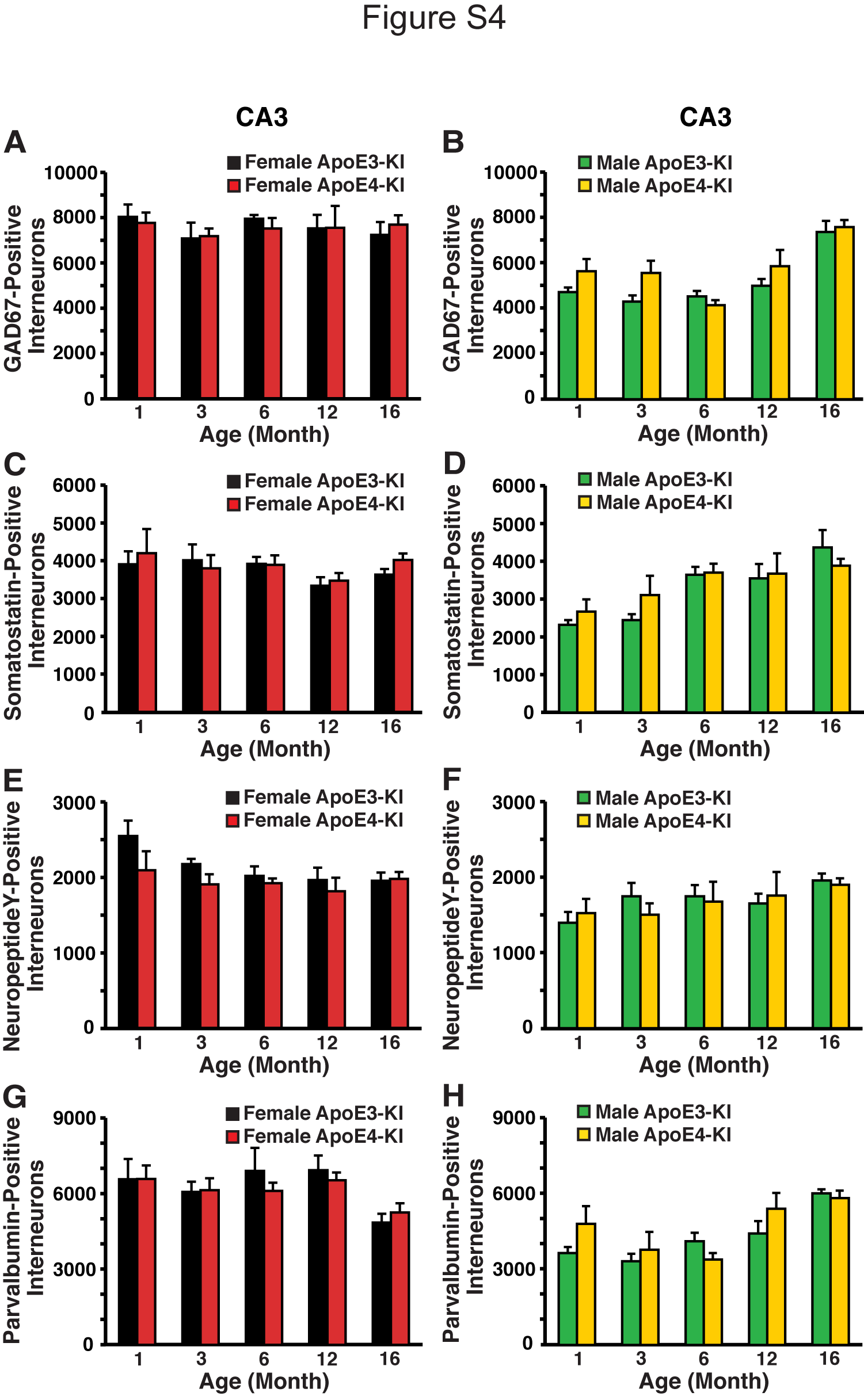

Supplement: Figure S4 — GABAergic interneuronal profiles in the CA3 change as a function of age, sex and apoE genotype. A–H, GABAergic interneurons in the CA3 positive for GAD67 (A, B), somatostatin (C, D), neuropeptide Y (E, F), and parvalbumin (G, H) in female (A, C, E, G) and male (B, D, F, H) apoE-KI mice at 1, 3, 6, 12, and 16 months of age (n = 6−12 mice per group). Results in histograms are presented as the total number of positive cells counted per brain. (TIF) [file pone.0053569.s004.tif]

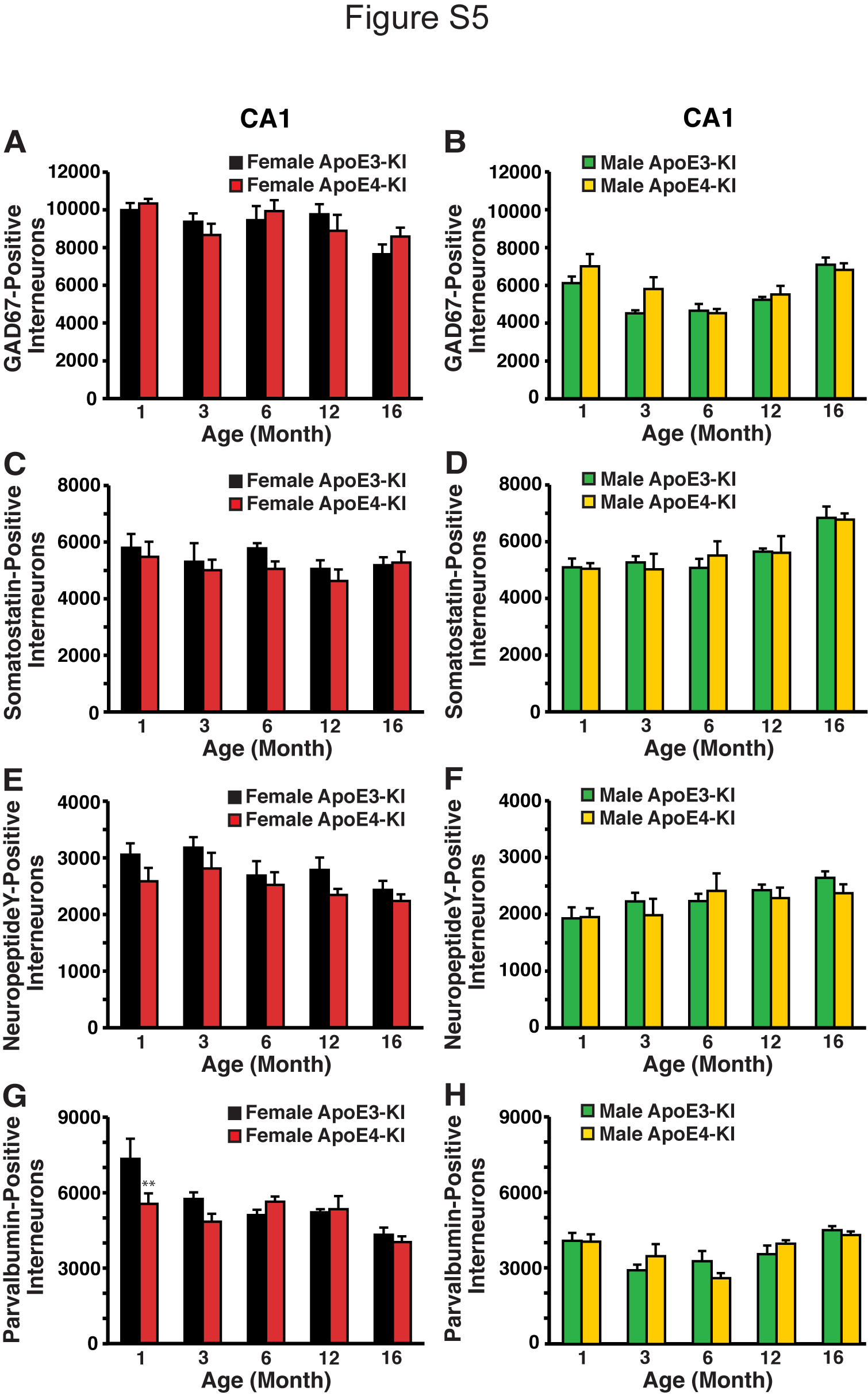

Supplement: Figure S5 — GABAergic interneuronal profiles in the CA1 change as a function of age, sex and apoE genotype. A–H, GABAergic interneurons in the CA1 positive for GAD67 (A, B), somatostatin (C, D), neuropeptide Y (E, F), and parvalbumin (G, H) in female (A, C, E, G) and male (B, D, F, H) apoE-KI mice at 1, 3, 6, 12, and 16 months of age (n = 6−12 mice per group). Results in histograms are presented as the total number of positive cells counted per brain. **p<0.01. (TIF) [file pone.0053569.s005.tif]

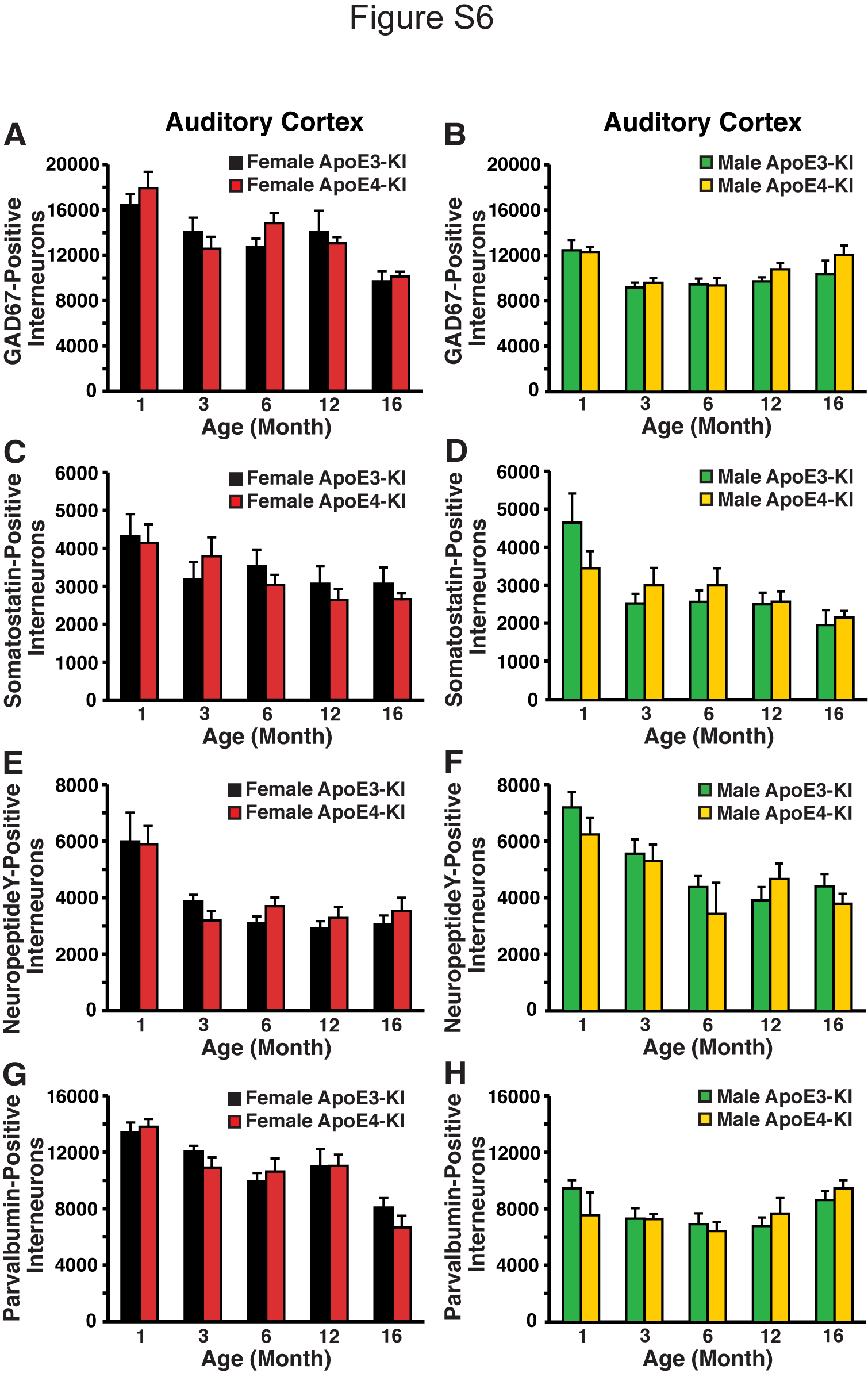

Supplement: Figure S6 — GABAergic interneurons in the auditory cortex change as a function of age, sex and apoE genotype. A–H, GABAergic interneurons in the auditory cortex positive for GAD67 (A, B), somatostatin (C, D), neuropeptide Y (E, F), and parvalbumin (G, H) in female (A, C, E, G) and male (B, D, F, H) apoE-KI mice at 1, 3, 6, 12, and 16 months of age (n = 6−12 mice per group). Results in histograms are presented as the total number of positive cells counted per brain. (TIF) [file pone.0053569.s006.tif]
